# Teaching Internal Medicine Residents to Critically Appraise the Role of Race in Pulmonary Function Testing

**DOI:** 10.15766/mep_2374-8265.11498

**Published:** 2025-02-20

**Authors:** Ananya Bhatia-Lin, Nirav Bhakta, Neha Deshpande, Laura Granados, Rosemary Adamson

**Affiliations:** 1 Third-Year Resident, Department of Internal Medicine, University of Washington School of Medicine; 2 Associate Professor, Department of Pulmonary, Critical Care, Asthma, and Sleep Medicine, University of California, San Francisco, School of Medicine; 3 Clinical Assistant Professor, Department of Internal Medicine, University of Washington School of Medicine; 4 Third-Year Fellow, Department of Pulmonary, Critical Care and Sleep Medicine, University of Washington School of Medicine; 5 Associate Professor, Department of Pulmonary, Critical Care and Sleep Medicine, University of Washington School of Medicine; Staff Physician, Pulmonary, Critical Care and Sleep Medicine Section, Veterans Affairs Puget Sound Healthcare System

**Keywords:** Pulmonary Function, Race, Race-Based Medicine, Racism in Medicine, Spirometry, Anti-racism, Health Equity, Online/Distance Learning, Pulmonary Medicine

## Abstract

**Introduction:**

Race-specific equations for spirometry reference values are one example of race-specific algorithms traditionally used in medicine. The American Thoracic Society now recommends use of race-neutral reference equations instead of race-specific equations. However, no published curricula on interpretation of spirometry using race-based compared to race-neutral reference equations exist. We developed a curriculum for internal medicine residents to address this gap and equip providers to interpret spirometry in a race-conscious fashion.

**Methods:**

An internal medicine resident and an attending in pulmonary medicine developed the curriculum and invited other experts to review and edit the material. The internal medicine resident delivered an hour-long, interactive, slide-based, didactic presentation during a weekly, residency-wide videoconference to 45 participants. The presentation included the following components: (1) history of spirometry and race, (2) race-specific equations, (3) race-neutral equations, and (4) clinical implications. The presentation opened with a clinical case and small-group discussions. We conducted pre- and posttest surveys; the posttest survey was designed using the Kirkpatrick model to assess reaction, learning, and anticipated behavioral change. Mean score differences were evaluated for level 2 questions using Cohen's *d* effect size.

**Results:**

Thirty-eight respondents completed the pretest survey, and 24 completed the posttest survey. Test scores significantly improved after session participation, with Cohen's *d* ranging from 0.27 to 1.17.

**Discussion:**

This curriculum was successful in engaging participants in critically appraising race-based interpretations of pulmonary function testing. The structure of the curriculum could be repurposed to create didactic content on other examples of race-based clinical algorithms.

## Educational Objectives

By the end of this activity, learners will be able to:

1. Name three reasons why race has been used as a factor in equations for pulmonary function test (PFT) predicted values.
2. List three potential reasons why lung function might be different in people of different races.
3. List three potential outcomes of the American Thoracic Society recommendation to use the global lung function race-neutral equations for patients of all races.
4. Describe the effects of using race-neutral rather than race-specific equations for PFT predicted values for a Black patient with dyspnea.

## Introduction

Many standardized tools for clinical decision-making utilize race-specific algorithms. The use of these race-based algorithms has significant clinical implications for the provision of care for patients from different racial groups. While the equations used for spirometry reference values were developed based on population studies demonstrating differences in lung function between racial/ethnic groups, they also rely on the assumption that race-specific differences are expected, due to the widespread belief in genetic differences between races. Genomic analyses have since demonstrated that racial categories are inadequate proxies for genetic variation.^1–4^

Advocates for anti-racism in medicine promote a shift from race-based medicine to race-conscious medicine. In race-conscious medicine, race is defined as a social and power construct, and racism is identified as a significant determinant of health outcomes.^5^ Race-specific equations for spirometry reference values are one example of race-based medicine that may result in clinical interpretations that vary depending on the patients’ race.

In 2023, the American Thoracic Society (ATS) recommended the use of race-neutral, instead of race-specific, reference equations for spirometry interpretation.^6^ Additionally, the ATS had previously recommended the use of Z-scores, rather than percentage predicted, as cutoffs to define abnormal pulmonary function tests (PFTs).^7^ This was an intentional shift away from race-based medicine. However, we suspect that many in the medical community were not aware of the widespread use of race-specific algorithms and thus are unaware of the clinical consequences of this change. Currently, there are only limited educational resources on race-specific interpretations of pulmonary function testing.^8,9^

In the wake of this decision, many providers need to adjust to the new guidelines. In addition to aiding in the diagnosis of obstructive and restrictive lung diseases, PFT results determine cutoffs for care eligibility. For Black patients, forced expiratory volume in first second of expiration and forced vital capacity Z-scores calculated using race-neutral equations are more likely to be under the lower limit of normal.^10^ This simultaneously increases disability payments and sensitivity to new pulmonary disease and decreases eligibility for certain thoracic surgeries and entry into certain occupations.

Despite these important implications for multiple aspects of patients’ lives, there are few standardized curricula for teaching PFTs to practitioners. A qualitative study across multiple levels of training identified unfamiliarity with spirometry as a major barrier to use.^11^ Innovations have been developed to teach pulmonary function testing in small-group workshops and through hands-on methods; however, these do not directly address the transition to Z-scores or how race impacts interpretation.^12,13^ Separately, curricula on the history of racism in medicine do exist, but most are targeted toward medical students and thus do not explore in detail the clinical implications of specific society guideline changes.^14,15^ While existing educational material may identify the existence of race-based medicine, there is a dearth of examples of curricula analyzing the impact of race-specific algorithms and their alternatives.

Thus, our educational innovation sought to address two recent transitions in interpretation of pulmonary function testing—the transition to Z-score interpretation and the anticipated transition to race-neutral reference equations. We developed a 45-minute, interactive, lecture-based presentation to cover the history of spirometry, interpretation through normal distributions and Z-scores, the history of race-specific normal distributions, and the impact of an anticipated transition to race-neutral reference equations. The target audience for this curriculum was internal medicine (IM) residents.

## Methods

An IM resident and a pulmonary and critical care medicine (PCCM) attending physician developed a PowerPoint presentation and accompanying script (Appendices A and B). The didactic began with a clinical case, then presented material in four sections: (1) the history of spirometry and race, (2) race-specific equations, (3) race-neutral equations, and (4) clinical implications. In the first section, we presented a timeline of race incorporated into lung function testing. In the second and third sections, we described how both race-specific and race-neutral equations were developed as well as critiques of these approaches. In the final section, we presented recent literature on the clinical implications of a shift to race-neutral equations.

We invited additional review of these materials by an IM attending and a PCCM fellow with experience in medical education as well as a PCCM attending from a different institution with expertise in PFT interpretation and race-neutral reference equations. We developed pre- and posttest surveys to analyze didactic efficacy utilizing the Kirkpatrick model for evaluation to assess participant reaction, learning, and impact on behavior.^16^ These surveys were piloted using cognitive interviewing on an IM resident. The University of Washington Institutional Review Board (IRB) granted IRB exemption from full review for the use of these surveys.

The IM resident presented the PowerPoint over Zoom videoconferencing during a residency-wide weekly didactic session. The presentation began with a clinical case discussion involving all participants, then transitioned to small-group virtual breakout rooms. Participants used the Global Lung Function Initiative online calculators to interpret spirometry utilizing race-specific and race-neutral equations (Appendix C).^17^ We encouraged large-group discussion in response to discussion questions through the Zoom chat tool. Utilizing dedicated didactic time ensured that residents were able to attend the conference without clinical distractions. Given that only a third of the residency program attended these didactic sessions at a given time, the presentation was recorded and disseminated through the residency program via a YouTube account. Asynchronous participants did not complete the pre- and posttest surveys and were not included in our study.

Participants completed pre- and posttest surveys immediately prior to and following the presentation. The pretest survey was composed of questions that addressed the Educational Objectives (Appendix D.) The posttest survey repeated the knowledge testing questions used in the pretest as well as two free-text boxes addressing learning points from the session and feedback (Appendix E). We developed a rubric to score pre- and posttest knowledge answers and calculated means and standard deviations for each question (Appendix F). We used Cohen's *d* to calculate effect size for each question.

## Results

Forty-five participants, consisting of IM residents (PGY 1-PGY 3), chief residents, and faculty, attended this talk. Of these participants, 37 completed the pretest survey, and 23 completed the posttest survey. When asked, “How do race-specific PFT equations differ from race-neutral equations?”, 78% of pretest respondents stated, “I don't know.” Following the presentation, 72% of posttest respondents correctly answered that race-specific PFT equations underdiagnose restriction in Black patients. Total scores for each question ranged from −2 for fully incorrect answers to 3 for fully correct answers based on the grading rubric (Appendix E). There was significant improvement in mean pre- and posttest scores for each of the knowledge assessment questions (Table, Figure).

**Table.**
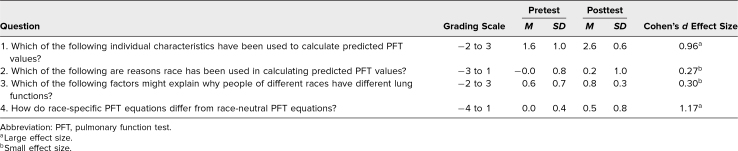
Pre- and Posttest Mean Scores, Standard Deviations, and Effect Sizes

**Figure.**
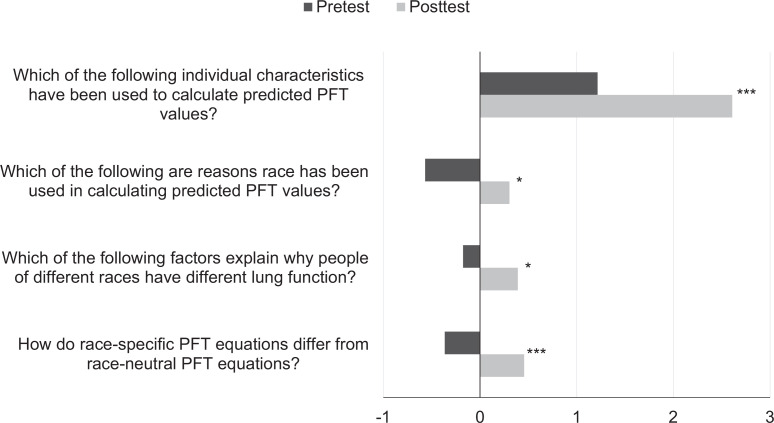
Pre- and posttests mean scores. Mean pre- and posttest scores for each question are represented by bars. Large effect size is denoted with ***; small effect size is denoted with *. Abbreviation: PFT, pulmonary function test.

All posttest survey respondents said they learned something new from this presentation and agreed with the statement “This [academic half day] presentation was engaging.” Respondents addressed Educational Objective 3 in their responses, stating,

The use of a race-neutral calculator can lead to changes in how you give care, both positively and negatively! can help identify disease in people who are Black earlier, but can also change outcomes/treatment options for things like lung cancer.

Respondents also stated that they had learned about Z-scores, about how race-based PFT interpretations were developed, and to think critically about using race-specific and race-neutral PFT reference values. While participants reflected dismay that there was “no clear answer,” they recognized that this is an evolving topic within pulmonology and there are no clear guidelines on how to address the limitations of race-neutral reference equations.

Our final question assessed impact on clinical practice for our participants, all of whom serve as primary care physicians while in residency. We asked, “Recently, the ATS recommended adoption of race-neutral PFT reference equations. How do you anticipate this impacting your primary care clinic patients?” Most participants demonstrated understanding of the nuances inherent in utilizing equations to define care, responding that “[race-neutral equations] may over diagnose some patients and under diagnose other patients, but [we] will still need to use multiple clinical data points to make decisions” and “remember the measurements are a tool, not the ultimate decision maker.” However, some respondents did not reflect the complexity intended, stating only, “Better diagnostic and management (less under diagnosing) for non-white patients” and “More equity!”

## Discussion

To our knowledge, this is the first example of a didactic addressing the history and current use of a race-based algorithm. It was delivered at a transition point for race-specific algorithms in pulmonary function testing—months after the ATS recommended use of race-neutral equations but before race-neutral equations had been adopted by the PFT labs at our institutions. The goal was to equip participants with the knowledge to critically appraise interpretations of pulmonary function using both race-specific and race-neutral equations and thus prevent unintentional harms from the inherent limitations of each of these algorithms.

This didactic was successful in engaging participants on the technicalities of pulmonary function interpretation based on participation during the presentation and responses to the posttest survey. Respondents provided positive feedback on the session, demonstrated objective improvement in posttest scores, and left free-text comments reflecting the Educational Objectives.

This educational module was designed for IM residents with knowledge of the basics of spirometry and diagnosis of obstructive and restrictive pulmonary pathophysiology. Even so, many participants were surprised to learn that Z-scores, rather than percentage predicted, are used to interpret spirometry, creating a barrier to engagement with initial discussion questions. We have therefore altered the slides and Appendix C to include percentage predicted values in the breakout room case in addition to Z-scores to facilitate early engagement with the topic. Our discussion was augmented by the presence of a PCCM attending who could answer nuanced questions regarding Z-scores and PFT interpretation given the recent changes in society guidelines.

Evaluation of this didactic session was limited by both survey development and feedback responses. Evaluation of pre- and posttest score changes could be improved by inclusion of demographic data as well as adjusting for the impact of prior curricula on race-based medicine. Furthermore, while we hope that this session will be viewed widely beyond the initial attendees, so far only 45 participants have completed the curriculum through a single session. Lastly, there was attrition between pre- and posttest completion, with only 62% of those who completed the pretest completing the posttest. Tests of knowledge retention over time were not completed.

In terms of the material covered in the didactic, we discussed the use of spirometry to define normal/abnormal patterns and how spirometry can suggest a restrictive ventilatory defect; however, the diagnosis of restriction requires measurement of lung volumes. We intentionally chose not to discuss lung volumes as we wanted to focus the session on the effects of using race-specific compared to race-neutral reference equations and currently there are no race-specific equations for lung volumes. The field of spirometry interpretation is evolving; therefore, other institutions adopting this curriculum may vary in whether they use race-specific or race-neutral reference equations, which might affect trainee answers to pre- and posttest questions. We anticipate future changes regarding the inclusion of race in spirometry, thresholds used to define normal and abnormal spirometry, and the way spirometry is used to inform clinical decision-making.

Ultimately, a truly race-conscious approach to interpreting lung function would go beyond spirometry and the limitations of our existing reference equations to identify how racism and structural determinants of health shape each patient's exposures to determinants of pulmonary function. This curriculum touches on some determinants that disproportionally affect minoritized racial populations. Future development is warranted to create curricula that focus wholly on how clinicians and trainees can best practice race-conscious diagnosis for pulmonary disease. This might include patient factors, such as zip code, Area Deprivation Index of childhood neighborhoods, and exposures to poverty.^18^

While this didactic curriculum focuses on race-based spirometry interpretation, the model for the presentation could be repurposed for any race-based clinical decision-making tool. One could follow a similar structure for glomerular filtration rate interpretation, cesarean section risk score calculators, or calculators for vascular disease to develop critical thinking on a topic. As the medical community moves away from race-based medicine and towards race-conscious medicine, curricula like this one will be essential to support educators and learners.

## Appendices


Untangling Race From Pulmonary Function Testing.pptxPresentation Script.docxBreakout Room Activity.docxPretest Survey.docxPosttest Survey.docxScoring Rubric.docx
*All appendices are peer reviewed as integral parts of the Original Publication.*
