# Supplementary material for: Teaching Internal Medicine Residents to Critically Appraise the Role of Race in Pulmonary Function Testing
Source: MedEdPORTAL. 2025 Feb 20;21:11498. doi: 10.15766/mep_2374-8265.11498 (PMC11839840; doi:10.15766/mep_2374-8265.11498)
Supplement: Supplementary file 1 — Untangling Race From Pulmonary Function Testing.pptxPresentation Script.docxBreakout Room Activity.docxPretest Survey.docxPosttest Survey.docxScoring Rubric.docx [file mep_2374-8265.11498-s001.zip › B. Presentation Script.docx]

*Utilize this script to deliver didactic curriculum. Noted here are times where you can pause for interaction from the audience. Also noted are the times when you might utilize one of the other appendices, ie the pre-test activity or the Breakout Room activity.*

**Title: Untangling Race from Pulmonary Function Testing**

| Segment | Script | Slide/Reference |
| --- | --- | --- |
| Introduction | *[Introduce yourself]* | Slide 1 |
|  | This talk explores how race changes our interpretation of pulmonary function. These are the learning objectives.  *[read through learning objectives]* | Slides 2 |
|  | In this talk, we will discuss the history of spirometry, the development of race-specific and race-neutral reference equations for spirometry, and clinical implications of a switch to race-neutral reference equations. | Slide 3 |
| *Pre-Test* | *[If using a pre-test, present the Pre-Test and provide 5 minutes for completion]* | Slide 4  **Appendix D. Pre-test survey** |
| *Breakout Room Activity* | *If using the Breakout Room Activity, we suggest following the structure below:*   - *Introduce the clinical case; consider asking an audience member to read the case* - *Poll Q – can use built-in polling software or ask people to participate in the chat* - *Open up breakout rooms. Provide Breakout Room document (Appendix C). After completion of activity, bring people back to discuss Question 2: “How does this change your interpretation/diagnosis?”* | Slide 5-7  **Appendix C. Breakout Room Activity** |
| History of Spirometry & Race | In order to understand why this patient's Z scores differed when using the two equations, we need to learn more about the history of spirometry and race. | Slide 8 |
|  | Let’s start in the 1700s. | Slide 9 |
|  | Thomas Jefferson listed potential lung structural differences among other defining physical attributes that set enslaved Black people apart from White people. He argued that these differences justified continued enslavement of Black people. | Slide 10 |
|  | It wasn’t until the 1840s that a spirometer was developed in the UK by John Hutchinson. This marks the first time lung function could be objectively measured in large groups of people. | Slide 11 |
|  | Following this, Samuel Cartwright, a Virginian physician and enslaver of Black people, built his own spirometer and used it to quantify the difference in lung function between Black enslaved people and free White people. He referenced Jefferson’s argument that Black people consume less oxygen to explain the 20% difference in lung function he calculated between Black enslaved people and similarly sized White people. | Slide 12 |
|  | At the end of the Civil War, Benjamin Gould wrote a book surveying the bodily characteristics of Black and White soldiers. In a chapter on lung function, Gould reports a difference in mean lung volume between black and white soldiers that persisted when adjusting for height. | Slide 13 |
|  | This data was used during Reconstruction and into the 1920s to argue for white supremacy, notably by Fredrick Hoffman to explain increased mortality in Black individuals after emancipation. | Slide 14 |
|  | Based on studies such as these, the medical establishment incorporated race-correction factors and then race-specific reference equations into pulmonary function testing. The interpretations of PFTs using these race-specific equations have been used for decades to make management decisions for patients.  In the modern era of precision medicine, new generations of clinicians absorbed the practice of using race-specific equations as being data-driven. There was not much thought about the reasons for categorizing people by race nor about the impact of doing so for pulmonary function testing. | Slide 15 |
| Race-Specific Equations | Now let’s examine race-specific reference equations in more detail. | Slide 16 |
|  | The equations that are currently most commonly used were developed by the Global Lung Function Initiative in 2012.  These reference equations were based on a heterogenous group of people who were not known to have respiratory disease and did not smoke. Their analysis resulted in equations being developed for four racial populations – Caucasian, North East Asian, South East Asian, and African American.  These curves demonstrate the population-specific regression analyses for FVC by age for men for each of these four groups. The mean fitted FVC is shown.  Unfortunately, a large swath of the world’s population, including the African continent and Indian subcontinent were excluded due to lack of data.  The GLI data was similar to prior data in that it showed the FVC of North East Asian men was 4-6% lower than white men and the FVC of African American men was 10-15% lower than white men. This data was used to develop the GLI race-specific reference equations for pulmonary function testing.  In 2017, the American Thoracic Society recommended using these population-specific GLI spirometry equations based on self-reported ancestry. Prior to that, other race/ethnic-specific equations or correction factors had been used. | Slide 17 |
|  | When an individual gets pulmonary function testing, their results are compared on a normal distribution matched to their age, sex, and height. This generates a Z-score based on the standard deviation of the population, that indicates how far away a value is from the mean. The Z score is used to determine when patients have abnormal lung function. Z scores below -1.645 are below the lower limit of normal and indicate likely abnormal lung function. Only 5% of the reference population is expected to have a Z-score lower than -1.645. | Slide 18 |
|  | The GLI race-specific reference equations match individual results to a race-specific normal distribution  Here's an example of what the normal distribution for black and white reference equations might look like based on the GLI 2012 data. | Slide 19 |
|  | These arrows represent Z score less than -1.645, which occurs at different FVCs for the two separate reference equations. | Slide 20 |
|  | Lets say someone has an absolute FVC value corresponding to this line. That means that their FVC would be considered within normal limits for the Black reference equation, but below the lower limit of normal for the White reference equation. | Slide 21 |
|  | Lets return to our GLI population. We see a difference between FVC between racial groups.  Samuel Cartwright and others concluded that the discrepancy in lung function between races indicated a biological or genetic difference between races.  However, we now know that race is not a proxy for genetics. It is in fact a social construct..  *[Ask participants the following question, request them to answer in the chat or verbally]* What other factors might be responsible for the differences in lung function?  *[Acknowledge short answers]* | Slide 22 |
|  | For example, we know that environmental factors like early childhood exposure to pollutants and infections worsen lung function. And we know that these exposures vary between racial groups. On average, Black people in the US are more frequently exposed to these factors.  Perhaps environmental and social exposures are enough to explain the lower lung function among Black and Asian populations | Slide 23 |
| Race-Neutral Equations | Including race in reference equations normalizes the idea of biologically innate differences in lung function between racial groups. This leads us to ignore the underlying structural inequities that are likely driving these differences instead of working to improve them.  Therefore, there has been a push in recent years to move towards the use of race-neutral equations which would “unmask” the health consequences of social inequities for certain populations. | Slide 24 |
|  | In 2023 the American Thoracic Society recommended the use of a race-neutral equation, GLI Global. This reference equation was developed using the same GLI 2012 dataset as the race-specific equations. One average equation for the whole dataset was developed by weighting the contribution from each of the groups so that smaller populations contributed more to the equation than they would have without the weighting.  However, this strategy still excludes Africa, India, and South America and critics note that the these racial categories were chosen without a clear justification. | Slide 25 |
| Clinical Implications | This is a complex issue. While we cannot anticipate all the consequences of switching to race-neutral equations, let’s explore some of the clinical implications. | Slide 26 |
|  | This study compared the use of race-specific versus race-neutral reference equations for Black and White patients.  In these graphs, the number of individuals categorized as having obstructive, restrictive, nonspecific and normal PFTs is defined by the width of the green and red lines. Lung function interpretation changes when race-neutral equations are used instead of race-specific equations – the green lines indicate resolution of respiratory impairment and the red lines indicate new respiratory impairment.  For White individuals the race-neutral equations mostly led to resolution of nonspecific pulmonary impairments, with some results being re-classified as obstructive. For Black individuals, the biggest change was in the number of “Normal” individuals reclassified as having a restrictive impairment – almost 10% of the cohort studied. | Slide 27 |
|  | The same study showed that application of race-neutral reference equations led to re-classification of the severity of disease for both Black and White patients. For Black patients, there was an increase in severity of disease.  This study illustrates the significant interpretation impact of switching from race-specific to race-neutral PFT equations, which will be most profound for Black individuals. | Slide 28 |
|  | The implications of this are complicated; you can imagine situations in which race-neutral equations could alleviate health disparities, and situations where race-neutral equations could worsen health disparities.  *[Question for the Chat]* What are some possible effects of race-neutral PFTs?  *[Acknowledge answers]* | Slide 29 |
|  | Race-neutral equations are likely to increase the number of Black and Asian people who are evaluated for ILD and who are eligible for treatment. However, using race-neutral equations may decrease the number of Asian and Black people who are eligible for lung cancer resection. | Slide 30 |
|  | This study demonstrates the impact of reference equations on lung cancer surgery recommendations  In this study, 225 thoracic surgeons were given a clinical vignette of a 71 y/o woman with a right upper lobe non-small cell lung cancer. They were randomized to 1 of 3 groups receiving a different percent predicted post-operative FEV1 value. This value is used in clinical guidelines for lung cancer treatment recommendations.  While the value of FEV1 in liters was the same, the percent predicted varied depending on reference equation used. Surgeons either received values calculated with the GLI Black reference equation, a race-neutral reference equation, or the GLI White reference equation. All percent predicted values were between 30 and 60%. Within this range, recommendations are not clear on the optimal management and so the surgical approach varies  The primary outcome was treatment recommendations. Surgeons recommended lobectomy, wedge resection, or radiation therapy. Lobectomy is considered the only curative treatment for lung cancer. The study found that surgeons randomized to the White reference equation were less likely to recommend lobectomy than those using the Black reference equation. This group was more likely to recommend wedge resection or radiation, which have worse clinical outcomes for patients. | Slide 31 |
| *Clinical Case* | Now, lets return to our clinical case.  *[Ask a participant to read the case conclusion]* | Slide 32-33 |
| Summary | In this talk, we reviewed the history of pulmonary function testing. We learned that race has been used in pulmonary function testing due to BOTH racist beliefs regarding the physiology of Black enslaved people AND studies like Benjamin Gould’s survey of soldiers and the GLI 2012. Arguments that Black people had intrinsically worse lung function were used to support slavery and argue against civil rights for Black people.  We also learned that exposures to stress, pollution, and poverty might explain racial differences in lung function. These exposures differ due to structural inequalities between races. Rather than accepting that certain racial groups have intrinsically worse health outcomes, we should move toward addressing the social, economic, and environmental drivers of those outcomes.  Finally, as we move from race-specific reference equations to race-neutral equations, patients of all races will be affected. Their access to care and eligibility for treatment may change. We will need to be thoughtful about how institutional change may impact individual patients based on their race. | Slide 34 |
|  | This list of recommended reading provides more information on race-specific reference equations | Slide 35 |
| *Post-test* | *[If using a post-test survey, present the Post-Test and provide 5 minutes for completion]* | Slide 36  **Appendix E. Post-test survey** |
