# Supplementary material for: Teaching Internal Medicine Residents to Critically Appraise the Role of Race in Pulmonary Function Testing
Source: MedEdPORTAL. 2025 Feb 20;21:11498. doi: 10.15766/mep_2374-8265.11498 (PMC11839840; doi:10.15766/mep_2374-8265.11498)
Supplement: Supplementary file 1 — Untangling Race From Pulmonary Function Testing.pptxPresentation Script.docxBreakout Room Activity.docxPretest Survey.docxPosttest Survey.docxScoring Rubric.docx [file mep_2374-8265.11498-s001.zip › C. Breakout Room Activity.docx]

*This is the handout used to guide the breakout room activity during the session. Provide this to the participants ahead of time or share it as a document in the chat. This activity should take 10 minutes total. Allow some time for technical challenges (i.e., total 15 minutes).*

**Breakout Room Handout:**

Case Presentation:

50 y/o man, 177cm tall, identifies as Black. Recently quit smoking after 50 pack-years. Initially presented to primary care clinic with shortness of breath with exertion.

He returns to clinic to discuss the result of the spirometry you obtained. You note that the spirometry results used the GLI African American reference equations.

Spirometry results using GLI African American Reference Equations:

|  | **Pre- bronchodilator** | **Post-bronchodilator** | **Z-score** | **% Predicted** |
| --- | --- | --- | --- | --- |
| FEV1 (L) | 2.6 | 2.7 | -1.259 | 81% |
| FVC (L) | 3.3 | 3.4 | -1.283 | 81% |
| FEV1/FVC | 0.788 | 0.794 | -0.062 | 99% |

Question 1: What pattern does the spirometry show?

1. Normal
2. Obstruction
3. Possible restriction
4. Not sure

Question 2: Use the GLI website (https://gli-calculator.ersnet.org/index.html) to calculate his Z-score and percent predicted using the GLI Race-Neutral Equations.

|  | **Pre-bronchodilator** | **Post-bronchodilator** | **Z-score** | **% Predicted** |
| --- | --- | --- | --- | --- |
| FEV1 (L) | 2.6 | 2.7 |  |  |
| FVC (L) | 3.3 | 3.4 |  |  |
| FEV1/FVC | 0.788 | 0.794 |  |  |

How does this change your interpretation/diagnosis?

Answers:

Question 1: What pattern does the spirometry show?

1. **Normal**
2. Obstruction
3. Possible restriction
4. Not sure

**The Z-scores are greater than -1.645 and therefore within normal limits for all values. Thus, there is no evidence of obstruction (normal FEV1/FVC ratio) and no suggestion of restriction (the vital capacity (FVC) is within normal limits).**

**Note that people may answer this question using percent predicted rather than Z-scores. However, you should reinforce the ATS recommendation to use Z-scores for interpretation. Z-scores are preferable because they are generated from the standard deviation of the normal distribution in question. Percent predicted does not account for the variation in the spread of the distributions at differing ages and heights. The result is 80-120% predicted does not correspond to the 95% confidence interval for most ages and heights. Using percent predicted creates age and height-associated biases when deciding who crosses a threshold.**

Question 2: Use the GLI website to calculate his Z score and percent predicted using the GLI Race-Neutral Equations.

|  | **Pre- bronchodilator** | **Post-bronchodilator** | **Z-score** | **% Predicted** |
| --- | --- | --- | --- | --- |
| FEV1 (L) | 2.6 | 2.7 | **-1.7** | **73%** |
| FVC (L) | 3.3 | 3.4 | **-1.7** | **73%** |
| FEV1/FVC | 0.788 | 0.794 | **-0.018** | **99%** |

How does this change your interpretation/diagnosis?

**Now the Z-score for the FVC is less than -1.645, this means that the vital capacity is less than the lower limit of normal. This suggests abnormal lung function. Specifically, this suggests that there may be a restrictive process responsible for decreased FVC. Restriction may be due to a parenchymal abnormality, such as an interstitial lung disease, or an abnormality of the thoracic cage, such as weakness or kyphoscoliosis.**
