# Supplementary material for: Teaching Internal Medicine Residents to Critically Appraise the Role of Race in Pulmonary Function Testing
Source: MedEdPORTAL. 2025 Feb 20;21:11498. doi: 10.15766/mep_2374-8265.11498 (PMC11839840; doi:10.15766/mep_2374-8265.11498)
Supplement: Supplementary file 1 — Untangling Race From Pulmonary Function Testing.pptxPresentation Script.docxBreakout Room Activity.docxPretest Survey.docxPosttest Survey.docxScoring Rubric.docx [file mep_2374-8265.11498-s001.zip › F. Scoring Rubric.docx]

**Scoring Rubric**

*This is the scoring rubric used to grade each question for both the pre- and posttest surveys. We assigned points for correct answers and negative points for incorrect answers, with no points for missed answers.*

**Q1:** Which of the following individual characteristics have been used to calculate predicted PFT values?

+1 point for “Age,” “Height,” or “Race”

-1 point for “Weight,” “Comorbidities”

No points for “I don’t know” or a missing correct answer

**Q2:** Which of the following are reasons race has been used in calculating predicted PFT values?

+1 point for “Studies have shown a difference in lung function between different racial groups”

-1 point for “Black individuals have larger thoracic cavities than White individuals,” “There are genetic differences in lung function between Black and White individuals,” or “Race-specific equations result in better outcomes for Black patients”

No points for “I don’t know” or a missing correct answer

**Q3:** Which of the following factors might explain why people of different races have different lung function?

+1 point for “Maternal stress,” “Genetic differences” or “Environmental pollutants”

-1 point for “Comorbidities” or “Dietary preferences”

No points for “I don’t know” or a missing correct answer

**Q4:** How do race-specific PFT equations differ from race-neutral PFT equations?

+1 point for “Race-specific PFT equations under-diagnose restrictive ventilatory defects in Black patients”

-1 point for all other answers: “Race-specific PFT equations over-diagnose all lung disease in Black patients,” “Race-neutral PFT equations over-diagnose restrictive ventilatory defects in White patients,” “Race-neutral PFT equations under-diagnose obstruction in Asian patients,” “Race-neutral PFT equations over-diagnose obstruction in Black patients.”

No points for “I don’t know” or a missing correct answer
