# Supplementary material for: Teaching Internal Medicine Residents to Critically Appraise the Role of Race in Pulmonary Function Testing
Source: MedEdPORTAL. 2025 Feb 20;21:11498. doi: 10.15766/mep_2374-8265.11498 (PMC11839840; doi:10.15766/mep_2374-8265.11498)
Supplement: Supplementary file 1 — Untangling Race From Pulmonary Function Testing.pptxPresentation Script.docxBreakout Room Activity.docxPretest Survey.docxPosttest Survey.docxScoring Rubric.docx [file mep_2374-8265.11498-s001.zip › D. Pretest Survey.docx]

Pretest:

*This is the pre-test survey provided to participants in the beginning of the session. We asked them to complete this survey after the session had started but prior to introducing the topic.*

1. My medical school training directly addressed the history of racism in modern medicine.
   - Yes
   - Somewhat
   - Not at all
   - Only as an optional component
2. My residency training directly addresses the history of racism in modern medicine.
   - Yes
   - Somewhat
   - Not at all
   - Only as an optional component
3. If a race-specific calculator is available, I generally use it to make clinical decisions.
   - Agree
   - Disagree
   - Other: __________________________
4. [Select all correct answers] Which of the following individual characteristics have been used to calculate predicted PFT values?
   - Age
   - Comorbidities
   - Height
   - Race
   - Weight
   - I don’t know
5. [Select all correct answers] Which of the following are reasons race has been used in calculating predicted PFT values?
   - There are genetic differences in lung function between Black and White individuals
   - Black individuals have larger thoracic cavities than White individuals
   - Studies have shown a difference in lung function between different racial groups
   - Race-specific equations result in better outcomes for Black patients
   - I don't know
6. [Select all correct answers] Which of the following factors might explain why people of different races have different lung functions?
   - Genetic differences
   - Environmental pollutants
   - Comorbidities
   - Maternal stress
   - Dietary preferences
   - I don't know
