# Supplementary material for: Teaching Internal Medicine Residents to Critically Appraise the Role of Race in Pulmonary Function Testing
Source: MedEdPORTAL. 2025 Feb 20;21:11498. doi: 10.15766/mep_2374-8265.11498 (PMC11839840; doi:10.15766/mep_2374-8265.11498)
Supplement: Supplementary file 1 — Untangling Race From Pulmonary Function Testing.pptxPresentation Script.docxBreakout Room Activity.docxPretest Survey.docxPosttest Survey.docxScoring Rubric.docx [file mep_2374-8265.11498-s001.zip › A. Untangling Race From Pulmonary Function Testing.pptx]

## Slide 1
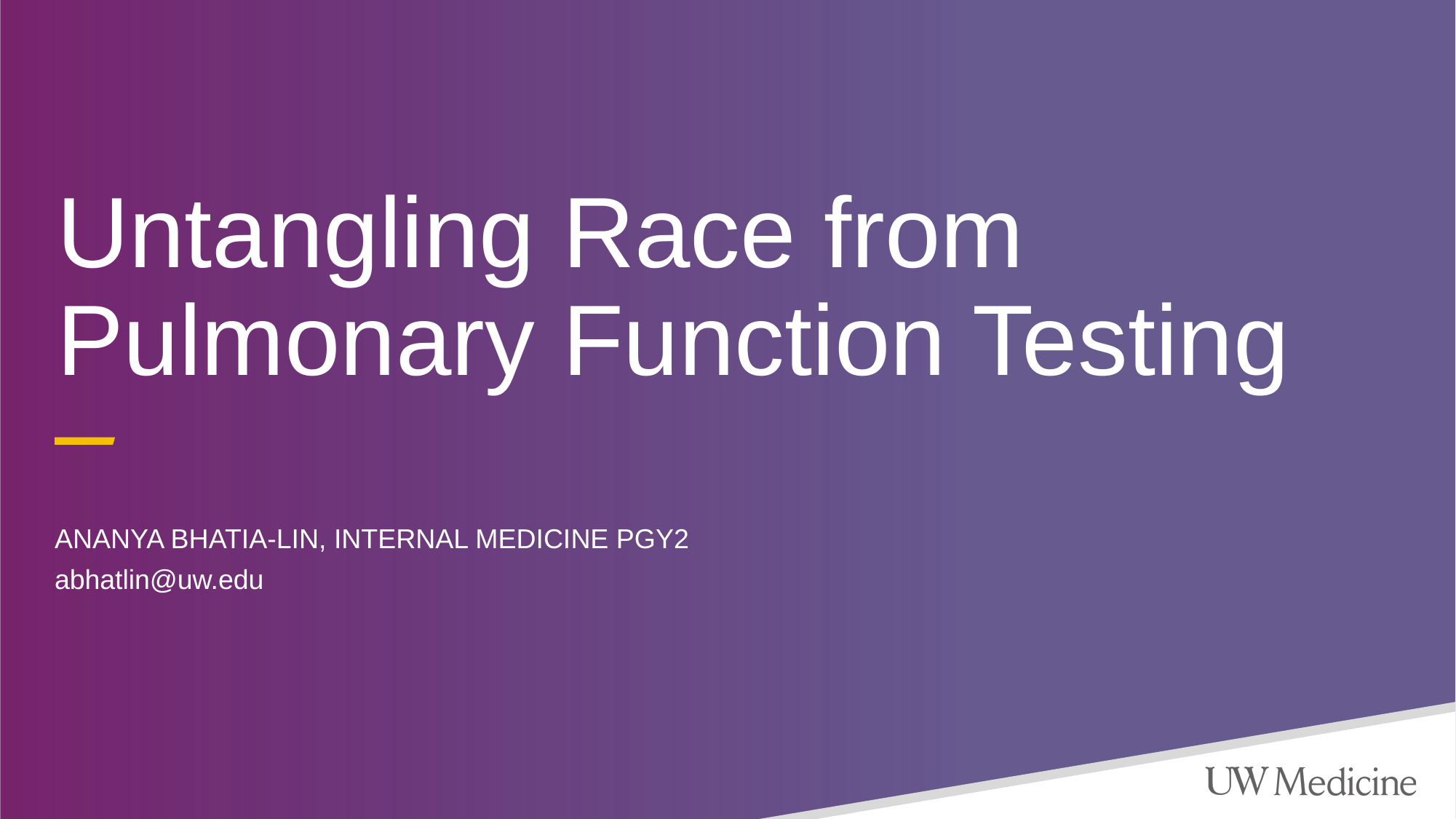

# Untangling Race from Pulmonary Function Testing
Ananya Bhatia-lin, internal medicine PGY2
abhatlin@uw.edu

## Slide 2
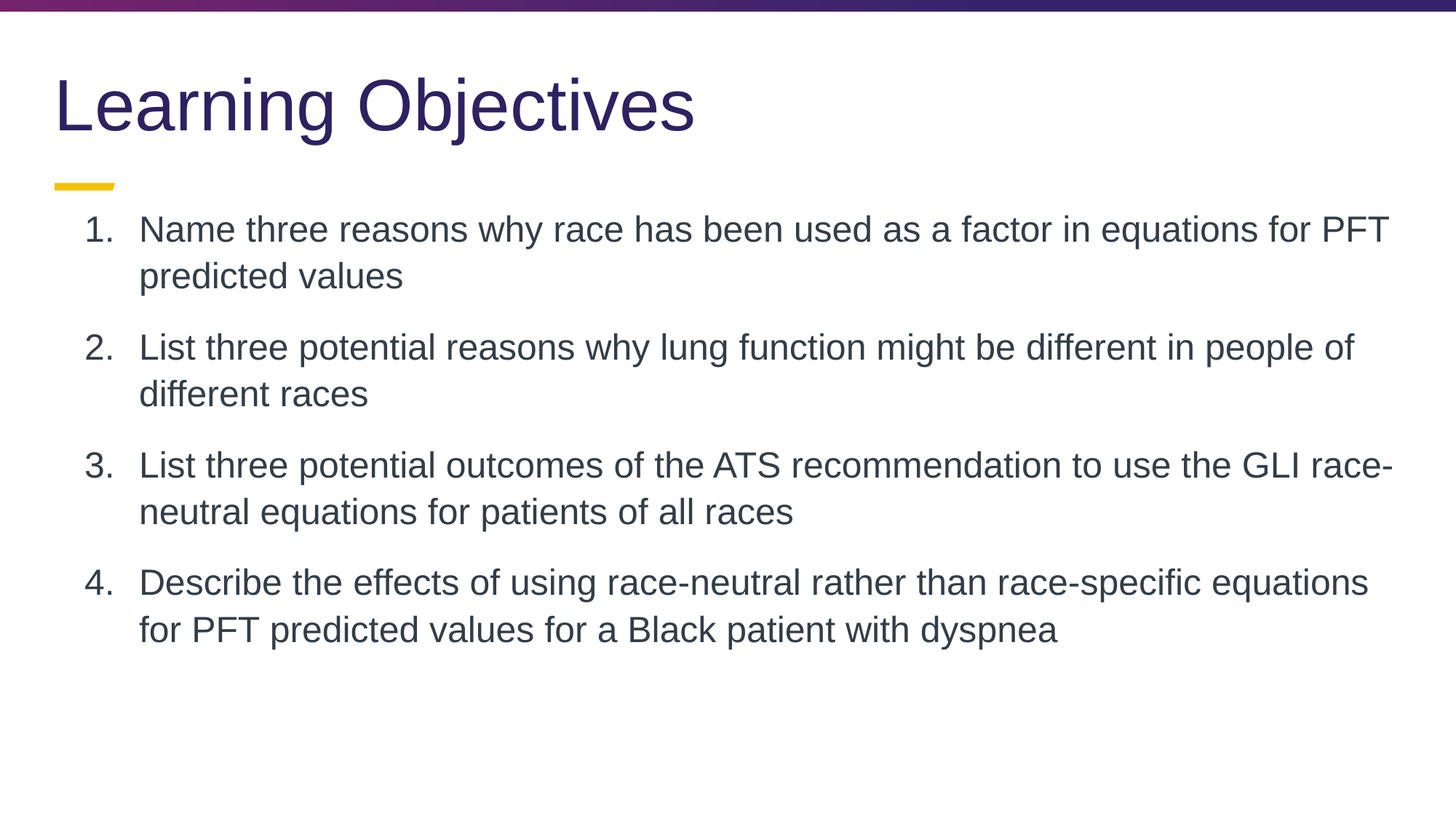

# Learning Objectives
Name three reasons why race has been used as a factor in equations for PFT predicted values
List three potential reasons why lung function might be different in people of different races
List three potential outcomes of the ATS recommendation to use the GLI race-neutral equations for patients of all races
Describe the effects of using race-neutral rather than race-specific equations for PFT predicted values for a Black patient with dyspnea

## Slide 3
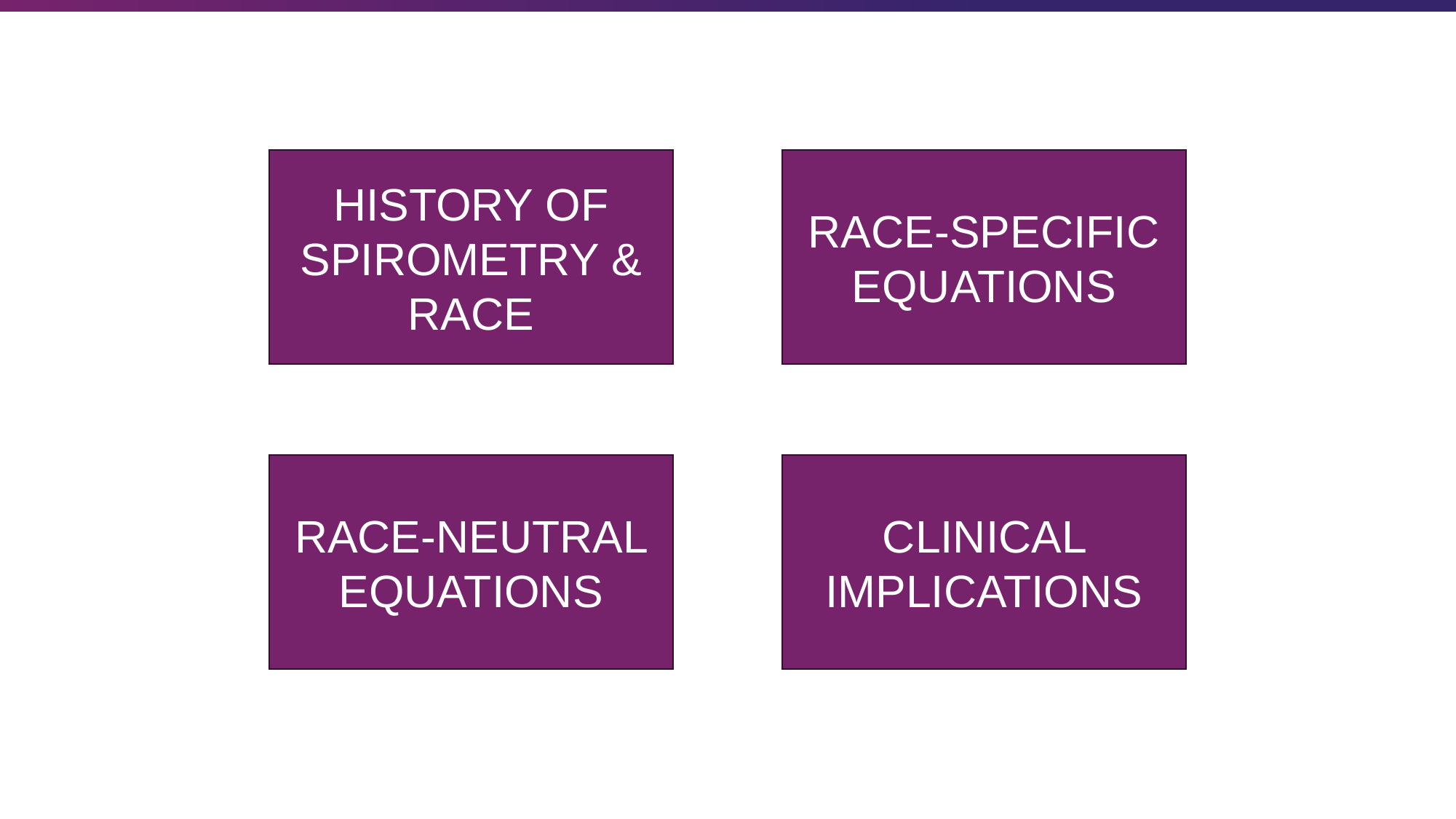

HISTORY OF SPIROMETRY & RACE
RACE-SPECIFIC EQUATIONS
RACE-NEUTRAL EQUATIONS
CLINICAL IMPLICATIONS

## Slide 4
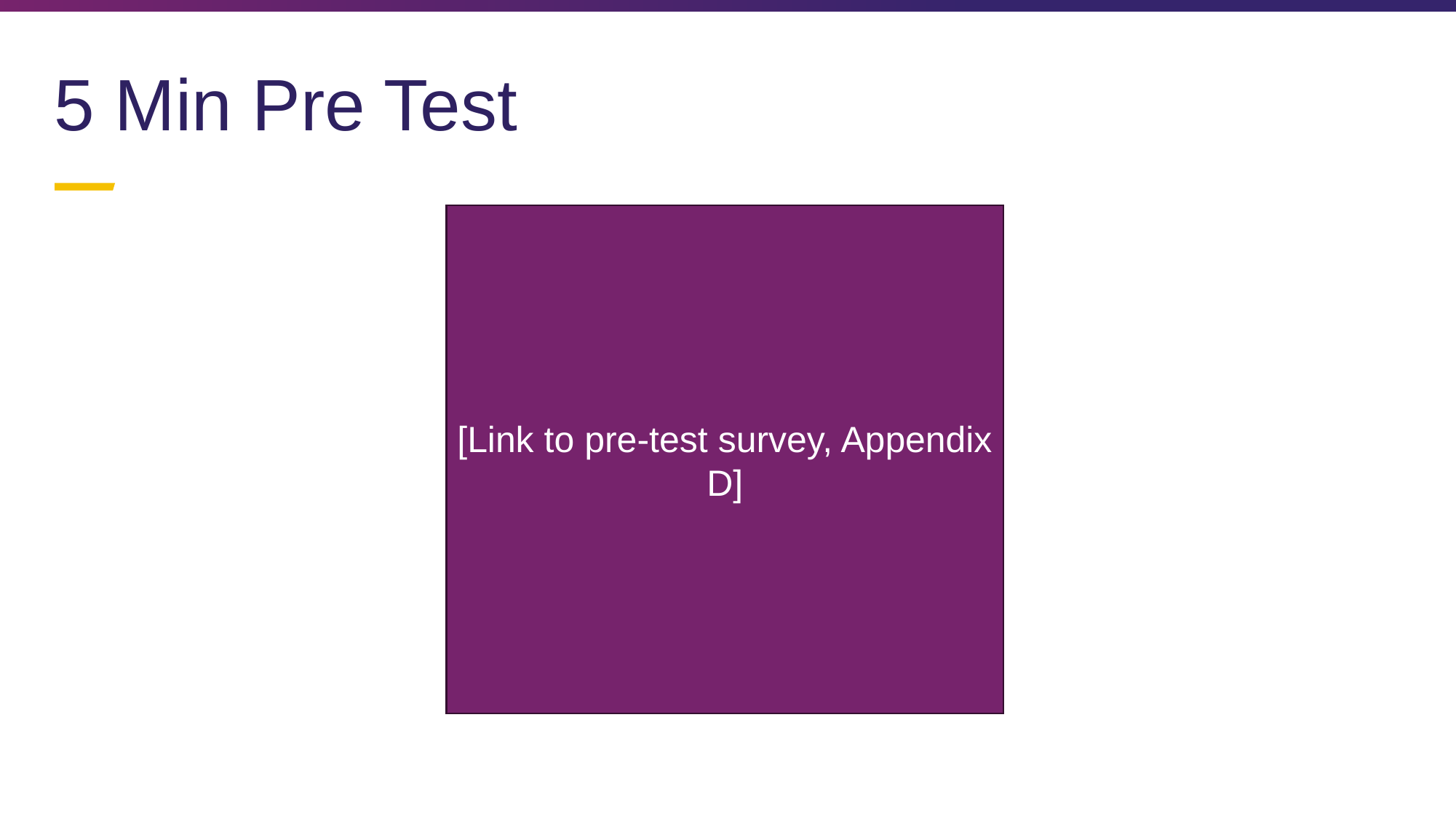

# 5 Min Pre Test
[Link to pre-test survey, Appendix D]

## Slide 5
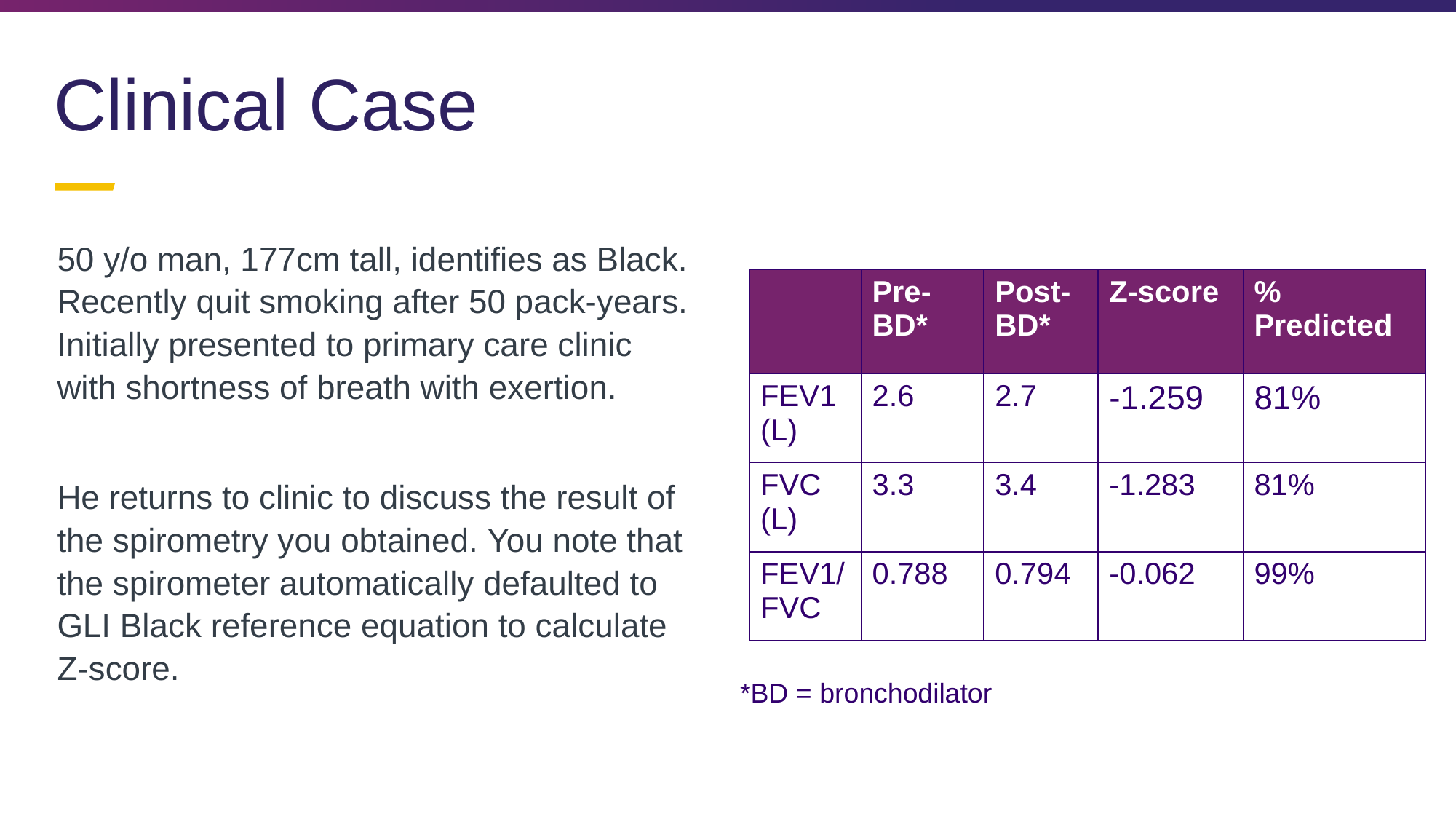

# Clinical Case
50 y/o man, 177cm tall, identifies as Black. Recently quit smoking after 50 pack-years. Initially presented to primary care clinic with shortness of breath with exertion.
He returns to clinic to discuss the result of the spirometry you obtained. You note that the spirometer automatically defaulted to GLI Black reference equation to calculate Z-score.
| | Pre- BD\* | Post-BD\* | Z-score | % Predicted |
| --- | --- | --- | --- | --- |
| FEV1 (L) | 2.6 | 2.7 | -1.259 | 81% |
| FVC (L) | 3.3 | 3.4 | -1.283 | 81% |
| FEV1/FVC | 0.788 | 0.794 | -0.062 | 99% |
*BD = bronchodilator

## Slide 6
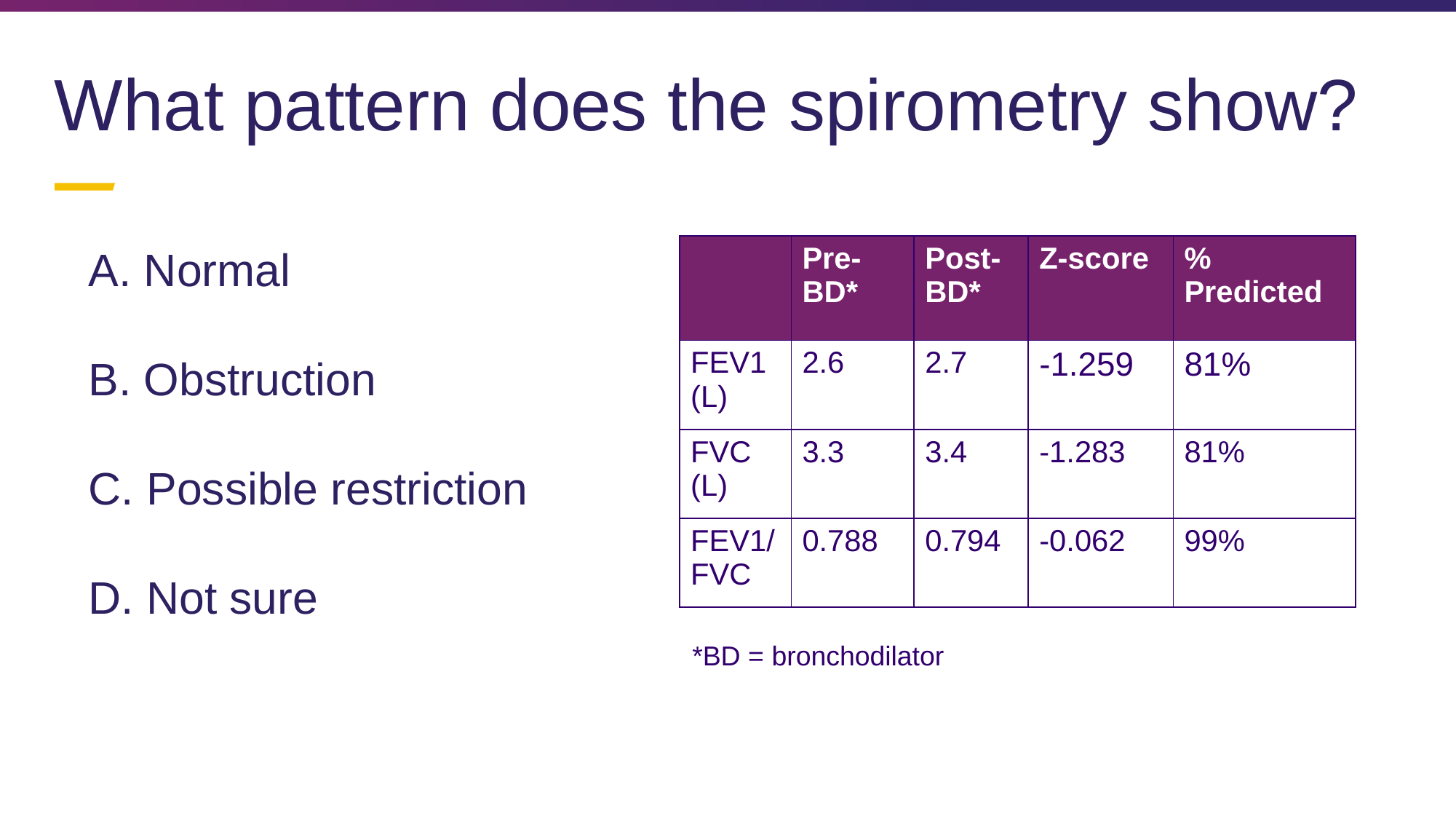

# What pattern does the spirometry show?
 Normal
 Obstruction
 Possible restriction
 Not sure
| | Pre- BD\* | Post-BD\* | Z-score | % Predicted |
| --- | --- | --- | --- | --- |
| FEV1 (L) | 2.6 | 2.7 | -1.259 | 81% |
| FVC (L) | 3.3 | 3.4 | -1.283 | 81% |
| FEV1/FVC | 0.788 | 0.794 | -0.062 | 99% |
*BD = bronchodilator

## Slide 7
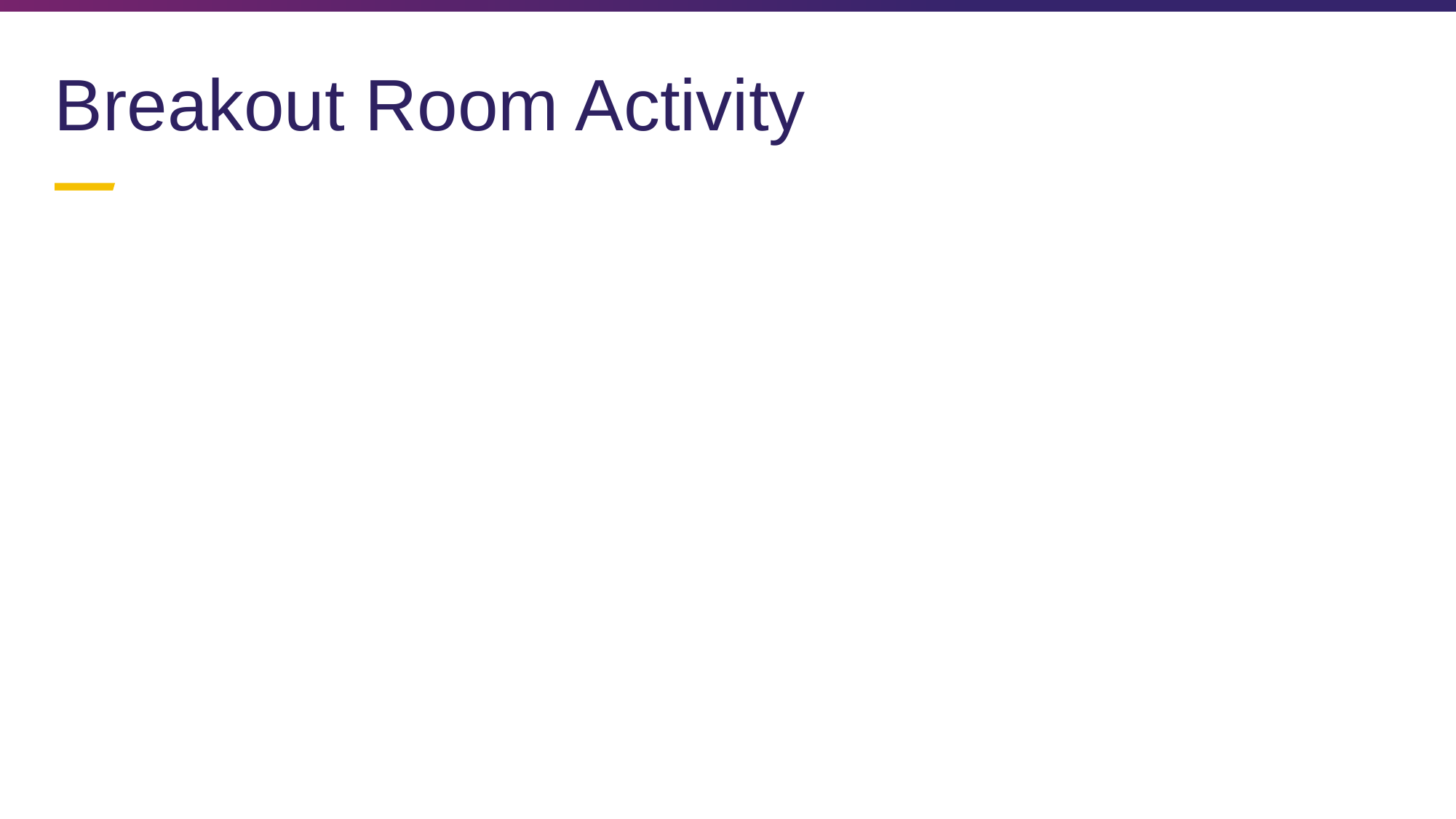

# Breakout Room Activity

## Slide 8
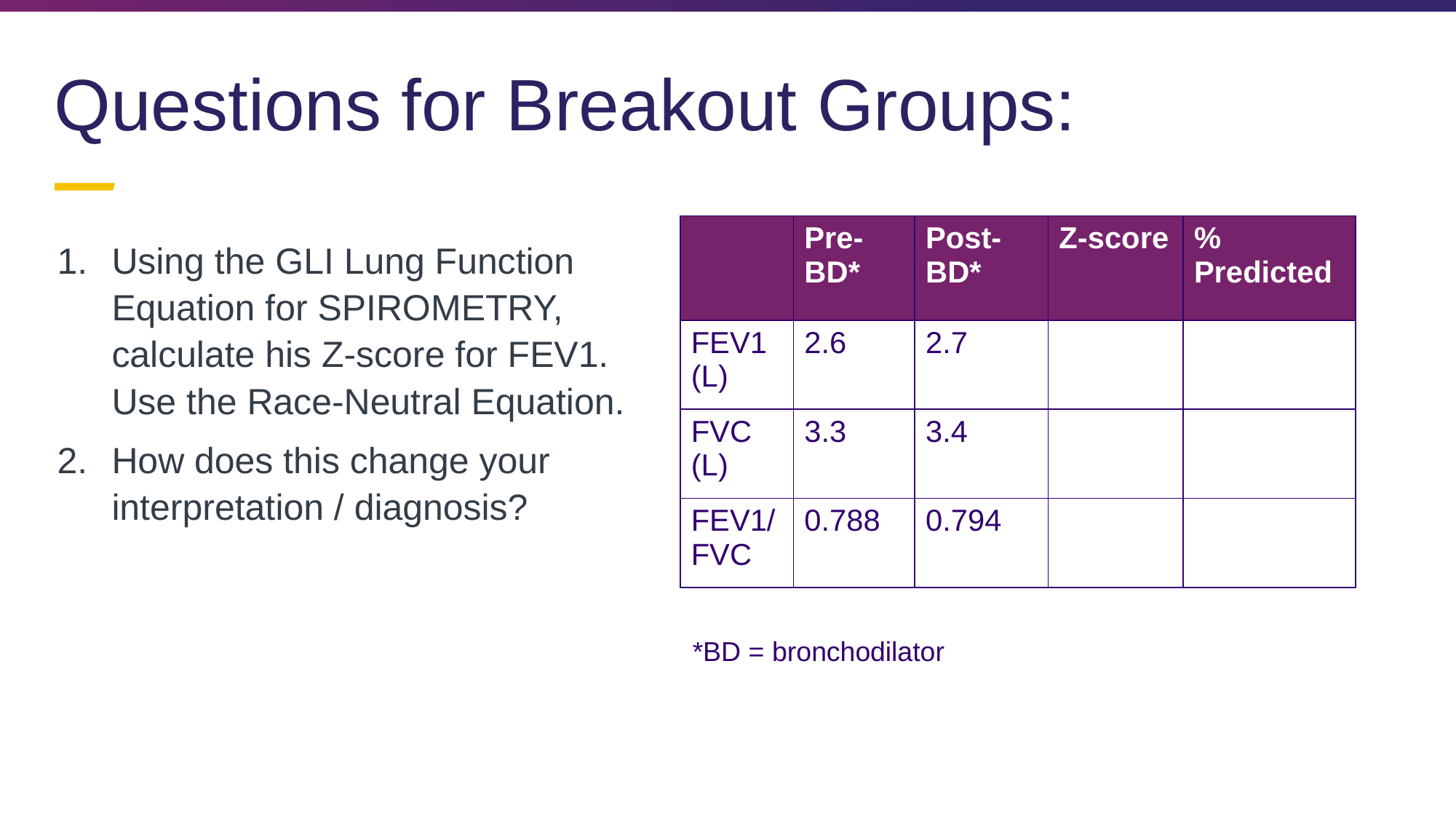

# Questions for Breakout Groups:
| | Pre- BD\* | Post-BD\* | Z-score | % Predicted |
| --- | --- | --- | --- | --- |
| FEV1 (L) | 2.6 | 2.7 | | |
| FVC (L) | 3.3 | 3.4 | | |
| FEV1/FVC | 0.788 | 0.794 | | |
Using the GLI Lung Function Equation for SPIROMETRY, calculate his Z-score for FEV1. Use the Race-Neutral Equation.
How does this change your interpretation / diagnosis?
*BD = bronchodilator

## Slide 9
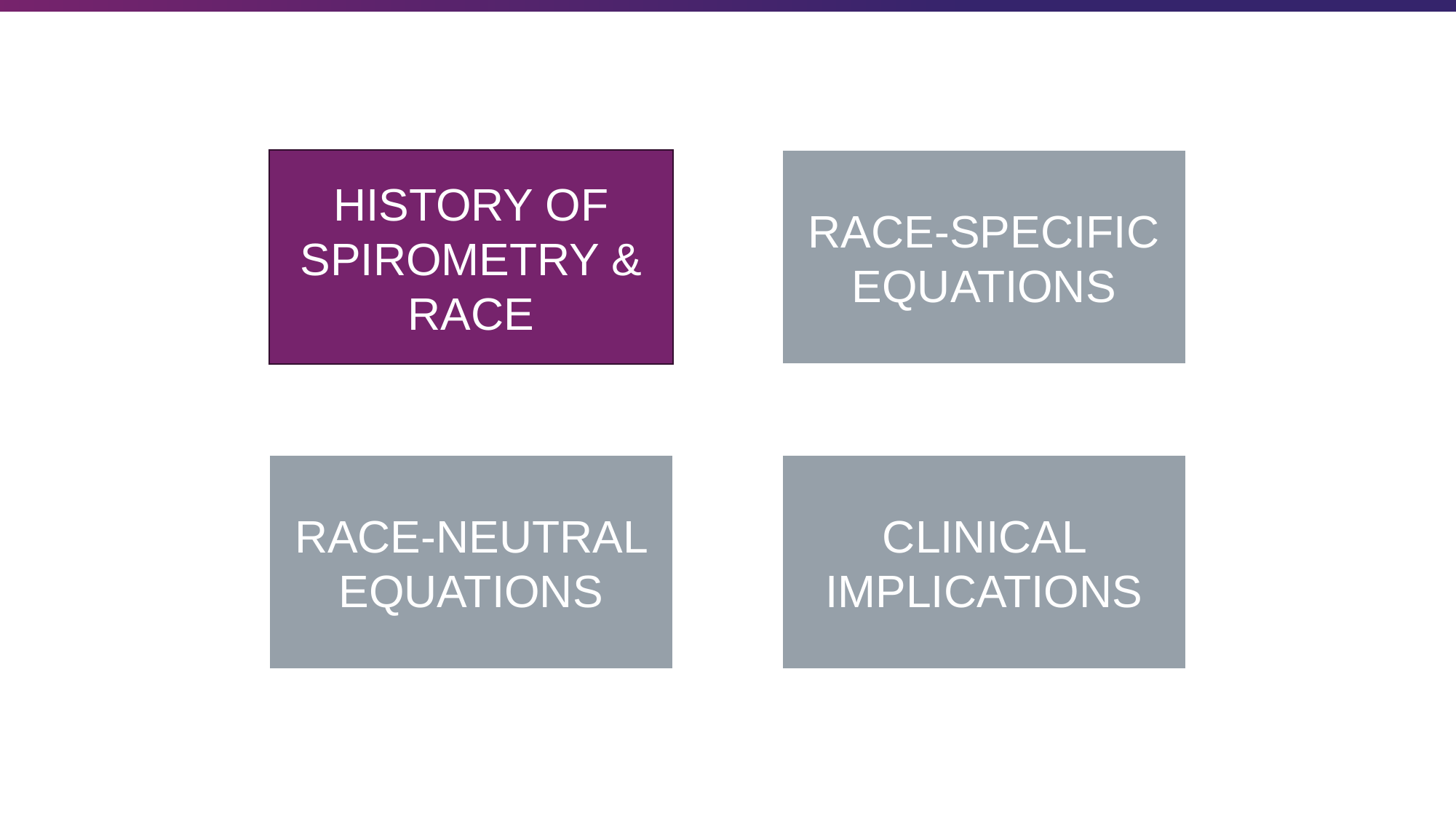

HISTORY OF SPIROMETRY & RACE
RACE-SPECIFIC EQUATIONS
RACE-NEUTRAL EQUATIONS
CLINICAL IMPLICATIONS

## Slide 10
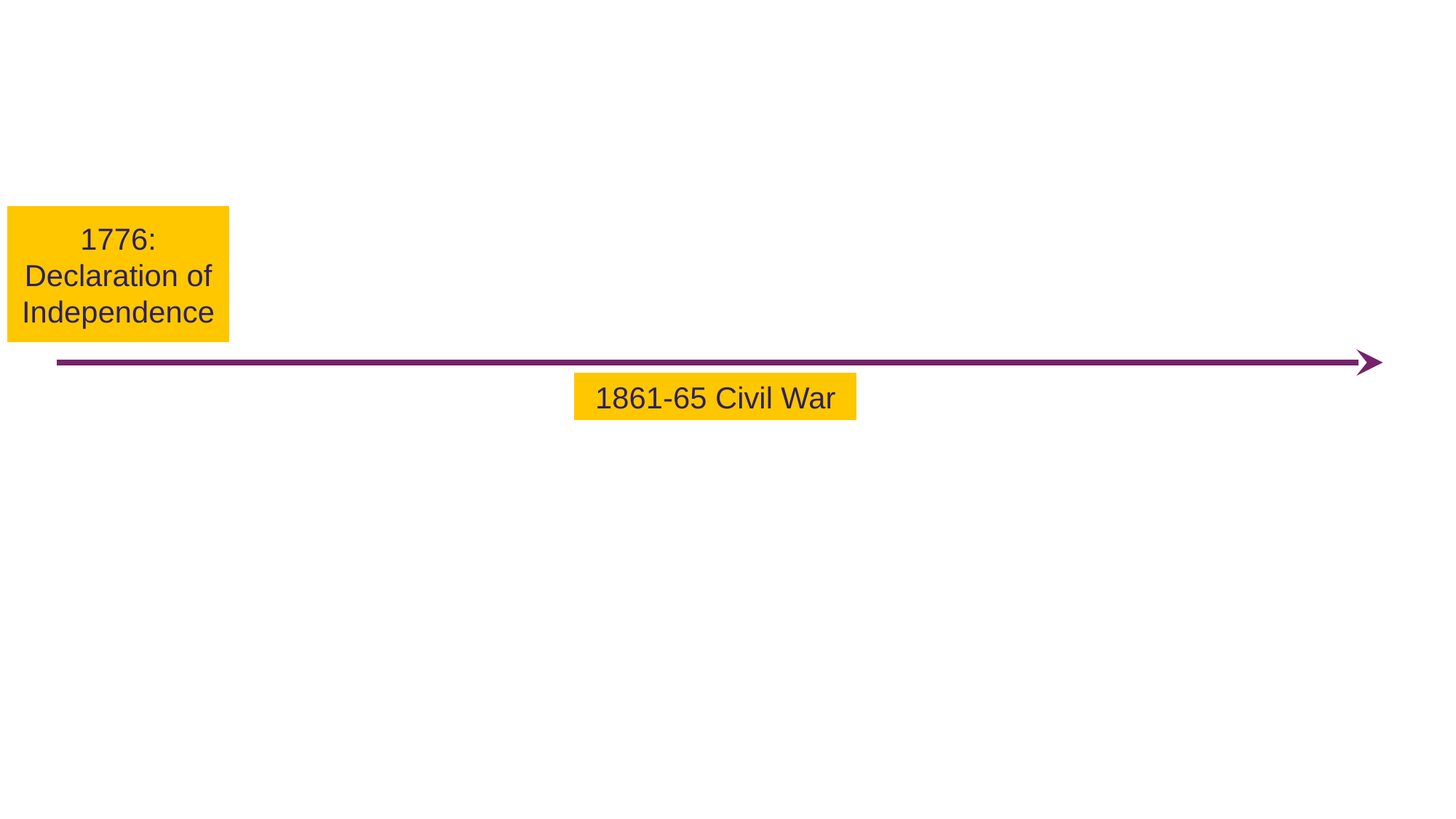

1776: Declaration of Independence
1861-65 Civil War

## Slide 11
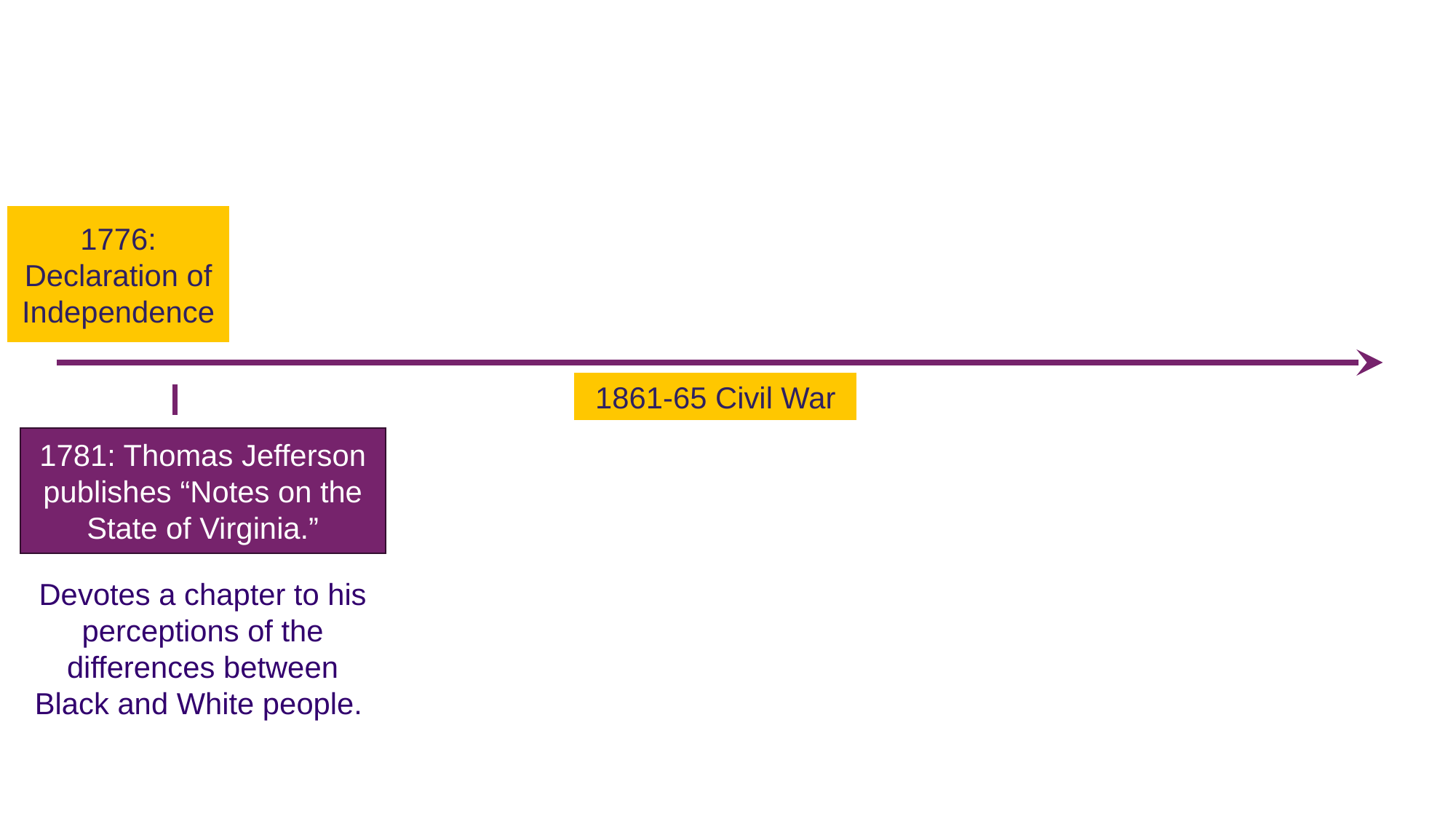

1776: Declaration of Independence
1861-65 Civil War
1781: Thomas Jefferson publishes “Notes on the State of Virginia.”
Devotes a chapter to his perceptions of the differences between Black and White people.

## Slide 12
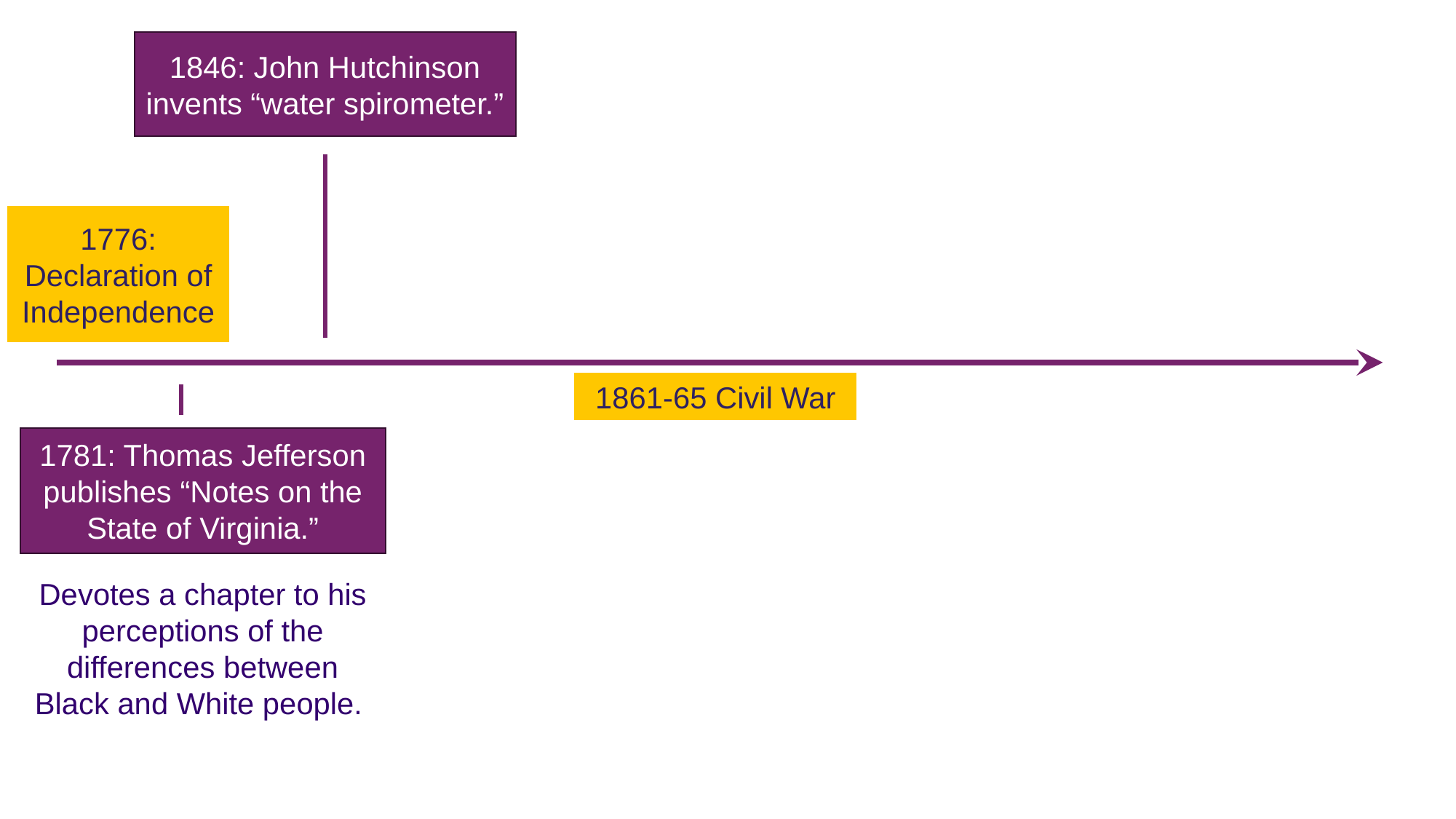

1846: John Hutchinson invents “water spirometer.”
1776: Declaration of Independence
1861-65 Civil War
1781: Thomas Jefferson publishes “Notes on the State of Virginia.”
Devotes a chapter to his perceptions of the differences between Black and White people.

## Slide 13
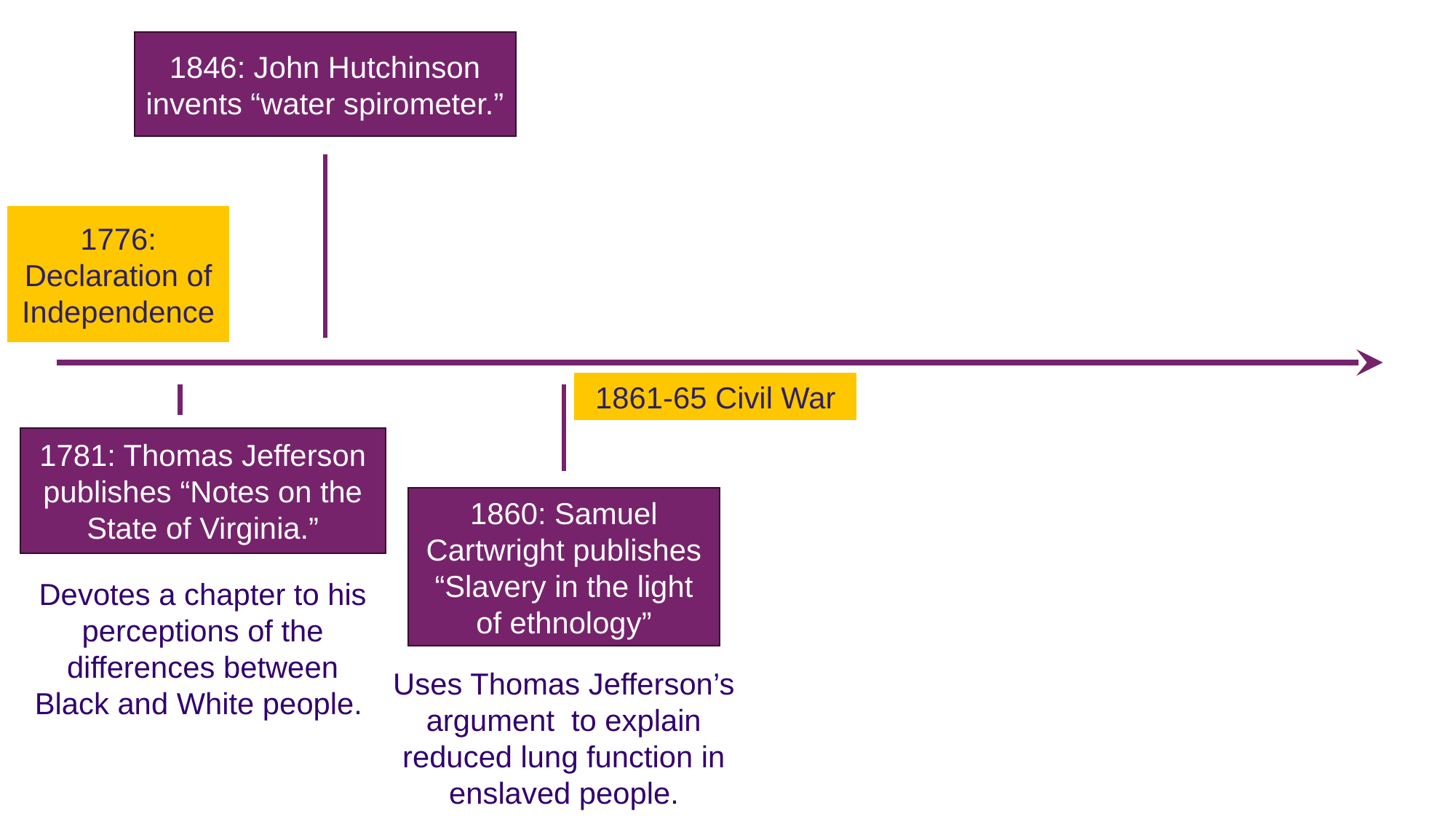

1846: John Hutchinson invents “water spirometer.”
1776: Declaration of Independence
1861-65 Civil War
1781: Thomas Jefferson publishes “Notes on the State of Virginia.”
1860: Samuel Cartwright publishes “Slavery in the light of ethnology”
Devotes a chapter to his perceptions of the differences between Black and White people.
Uses Thomas Jefferson’s argument to explain reduced lung function in enslaved people.

## Slide 14
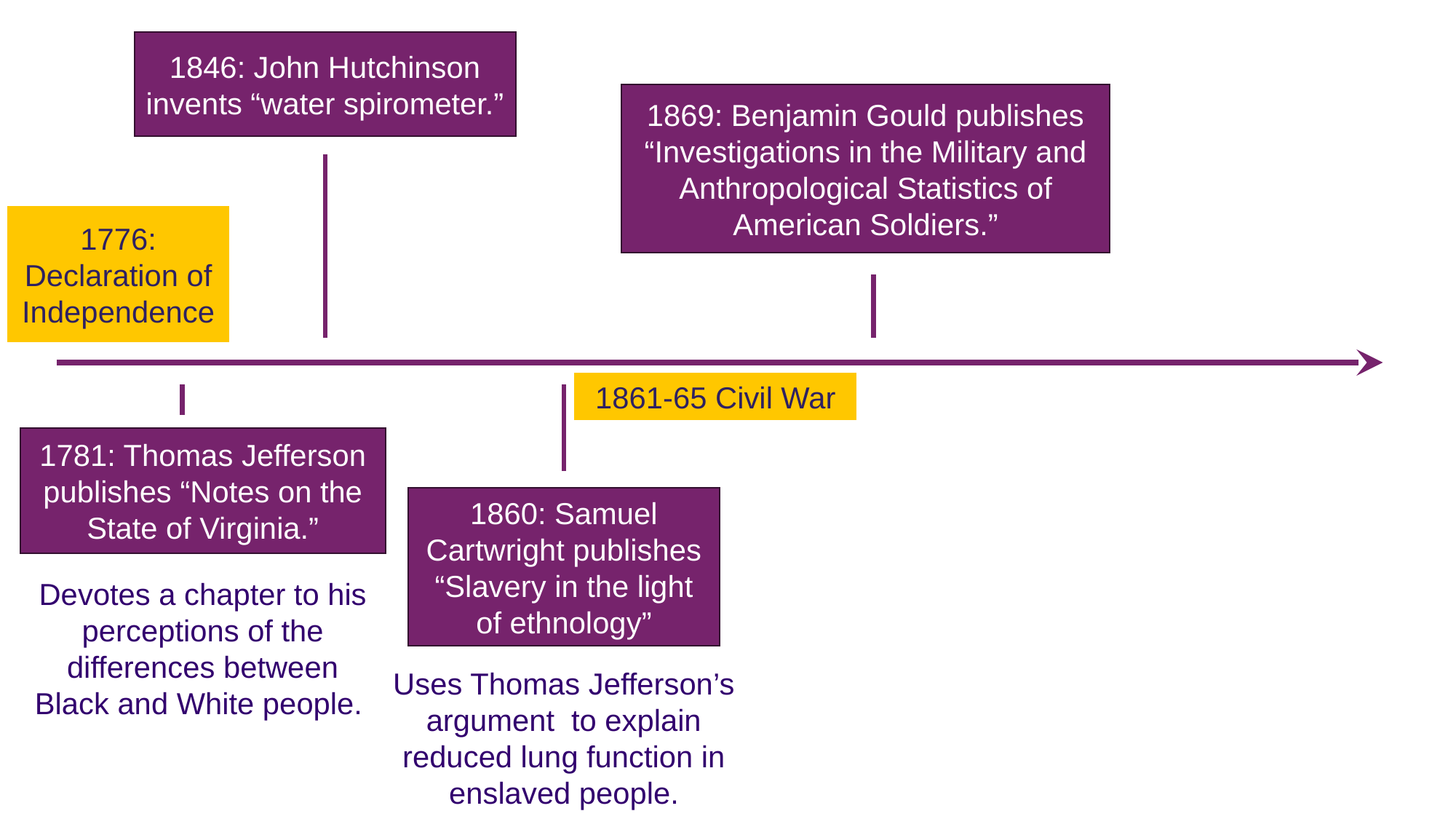

1846: John Hutchinson invents “water spirometer.”
1869: Benjamin Gould publishes “Investigations in the Military and Anthropological Statistics of American Soldiers.”
1776: Declaration of Independence
1861-65 Civil War
1781: Thomas Jefferson publishes “Notes on the State of Virginia.”
1860: Samuel Cartwright publishes “Slavery in the light of ethnology”
Devotes a chapter to his perceptions of the differences between Black and White people.
Uses Thomas Jefferson’s argument to explain reduced lung function in enslaved people.

## Slide 15
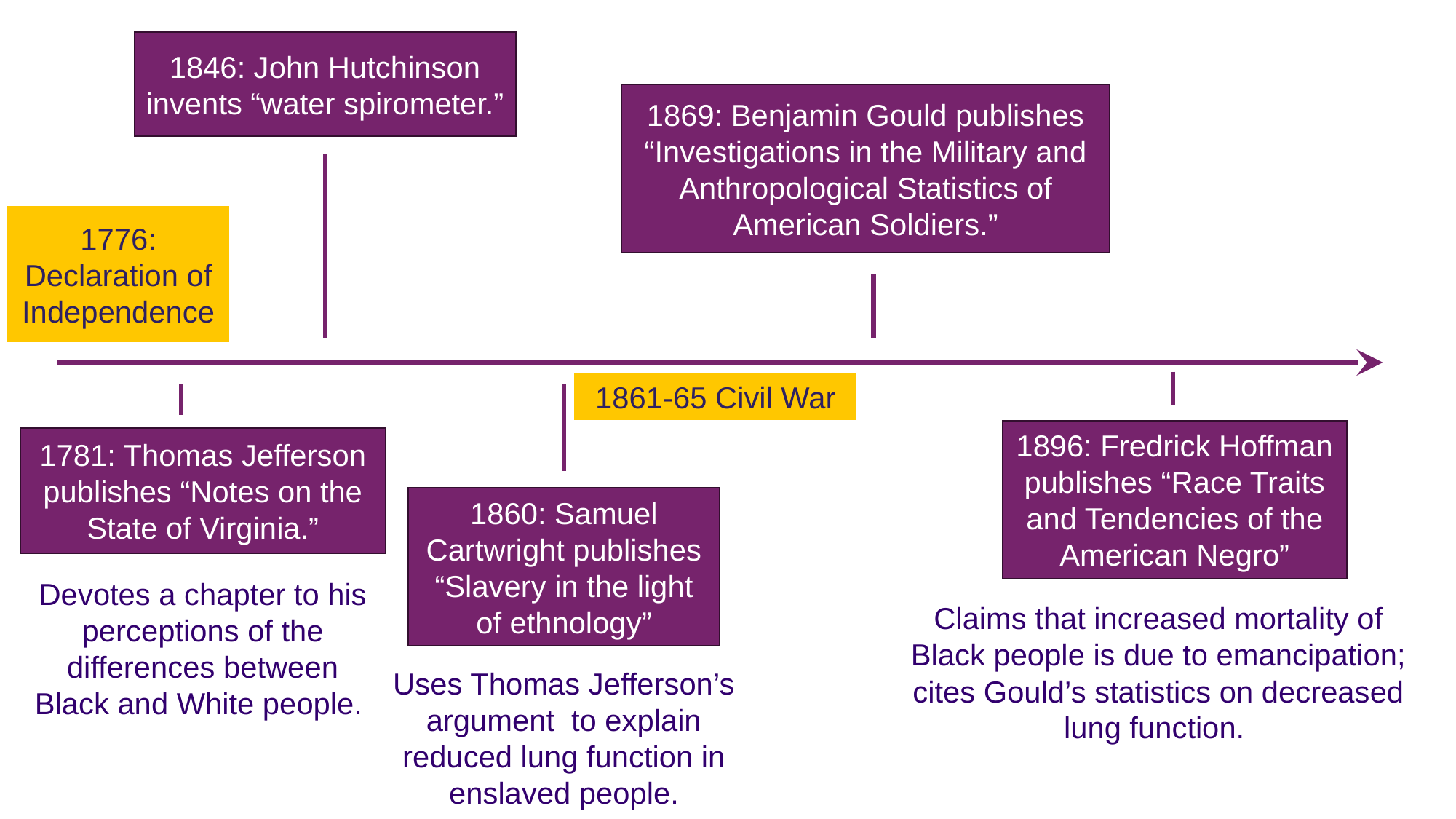

1846: John Hutchinson invents “water spirometer.”
1869: Benjamin Gould publishes “Investigations in the Military and Anthropological Statistics of American Soldiers.”
1776: Declaration of Independence
1861-65 Civil War
1896: Fredrick Hoffman publishes “Race Traits and Tendencies of the American Negro”
1781: Thomas Jefferson publishes “Notes on the State of Virginia.”
1860: Samuel Cartwright publishes “Slavery in the light of ethnology”
Devotes a chapter to his perceptions of the differences between Black and White people.
Claims that increased mortality of Black people is due to emancipation; cites Gould’s statistics on decreased lung function.
Uses Thomas Jefferson’s argument to explain reduced lung function in enslaved people.

## Slide 16
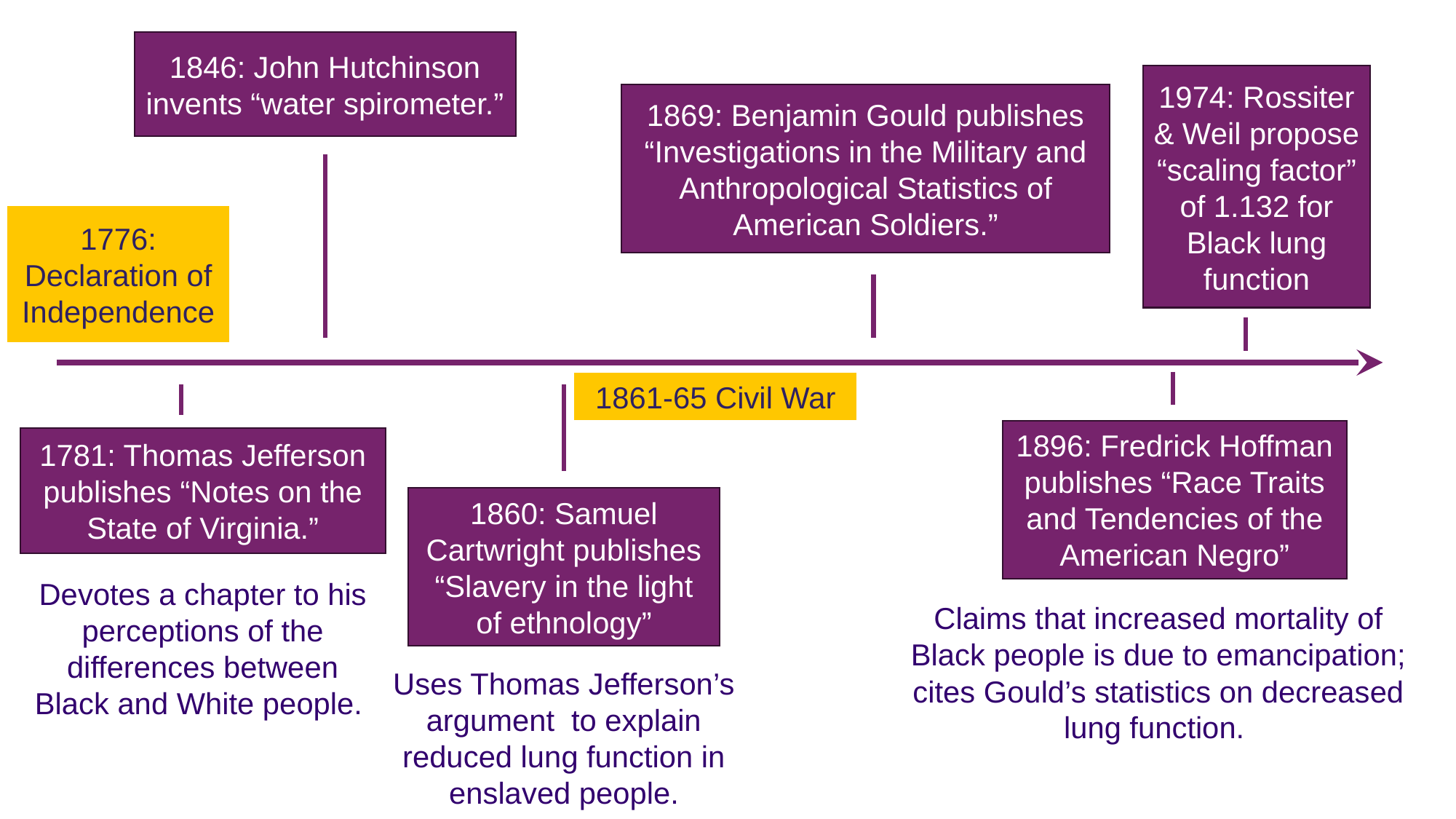

1846: John Hutchinson invents “water spirometer.”
1974: Rossiter & Weil propose “scaling factor” of 1.132 for Black lung function
1869: Benjamin Gould publishes “Investigations in the Military and Anthropological Statistics of American Soldiers.”
1776: Declaration of Independence
1861-65 Civil War
1896: Fredrick Hoffman publishes “Race Traits and Tendencies of the American Negro”
1781: Thomas Jefferson publishes “Notes on the State of Virginia.”
1860: Samuel Cartwright publishes “Slavery in the light of ethnology”
Devotes a chapter to his perceptions of the differences between Black and White people.
Claims that increased mortality of Black people is due to emancipation; cites Gould’s statistics on decreased lung function.
Uses Thomas Jefferson’s argument to explain reduced lung function in enslaved people.

## Slide 17
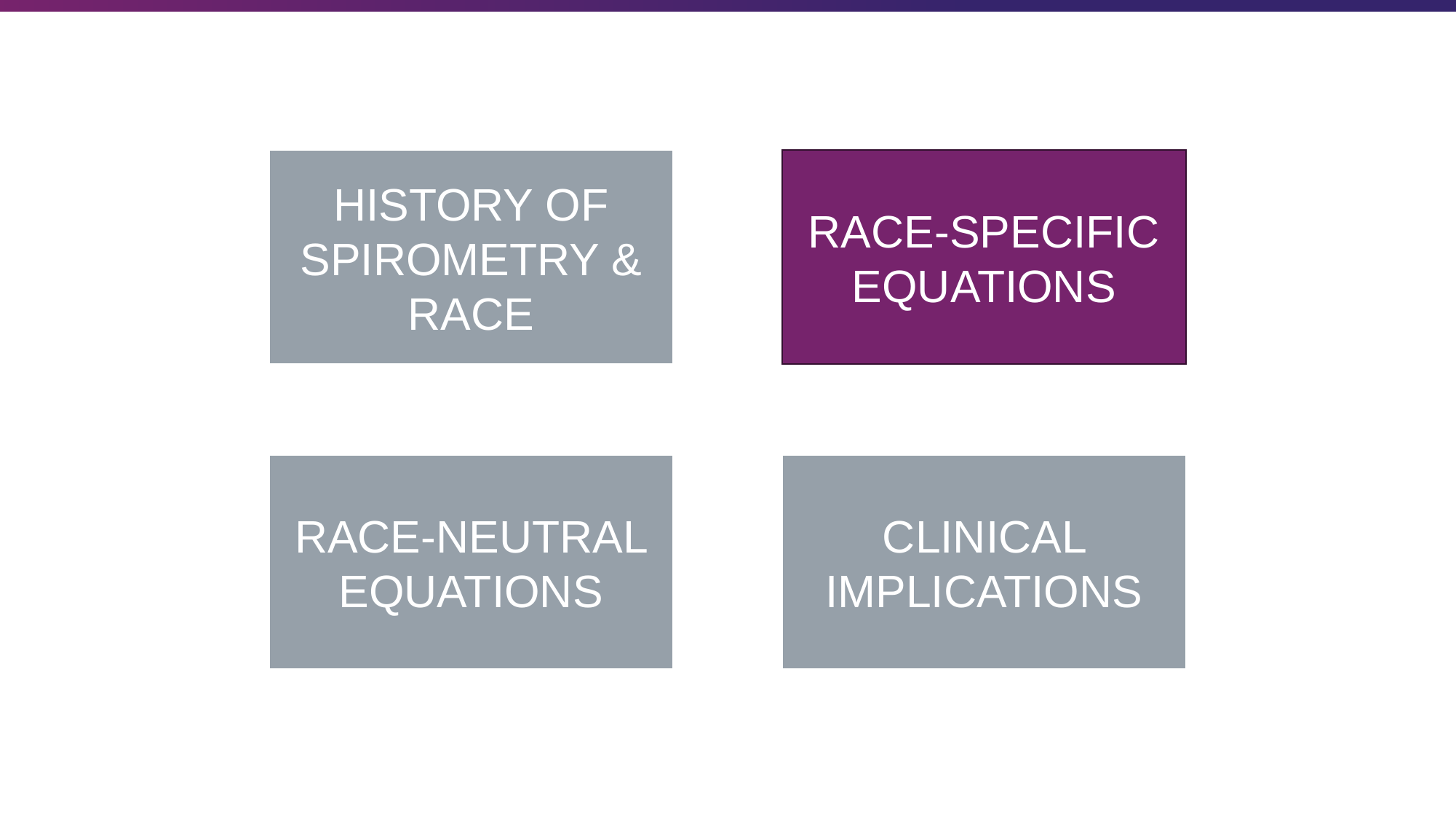

HISTORY OF SPIROMETRY & RACE
RACE-SPECIFIC EQUATIONS
RACE-NEUTRAL EQUATIONS
CLINICAL IMPLICATIONS

## Slide 18
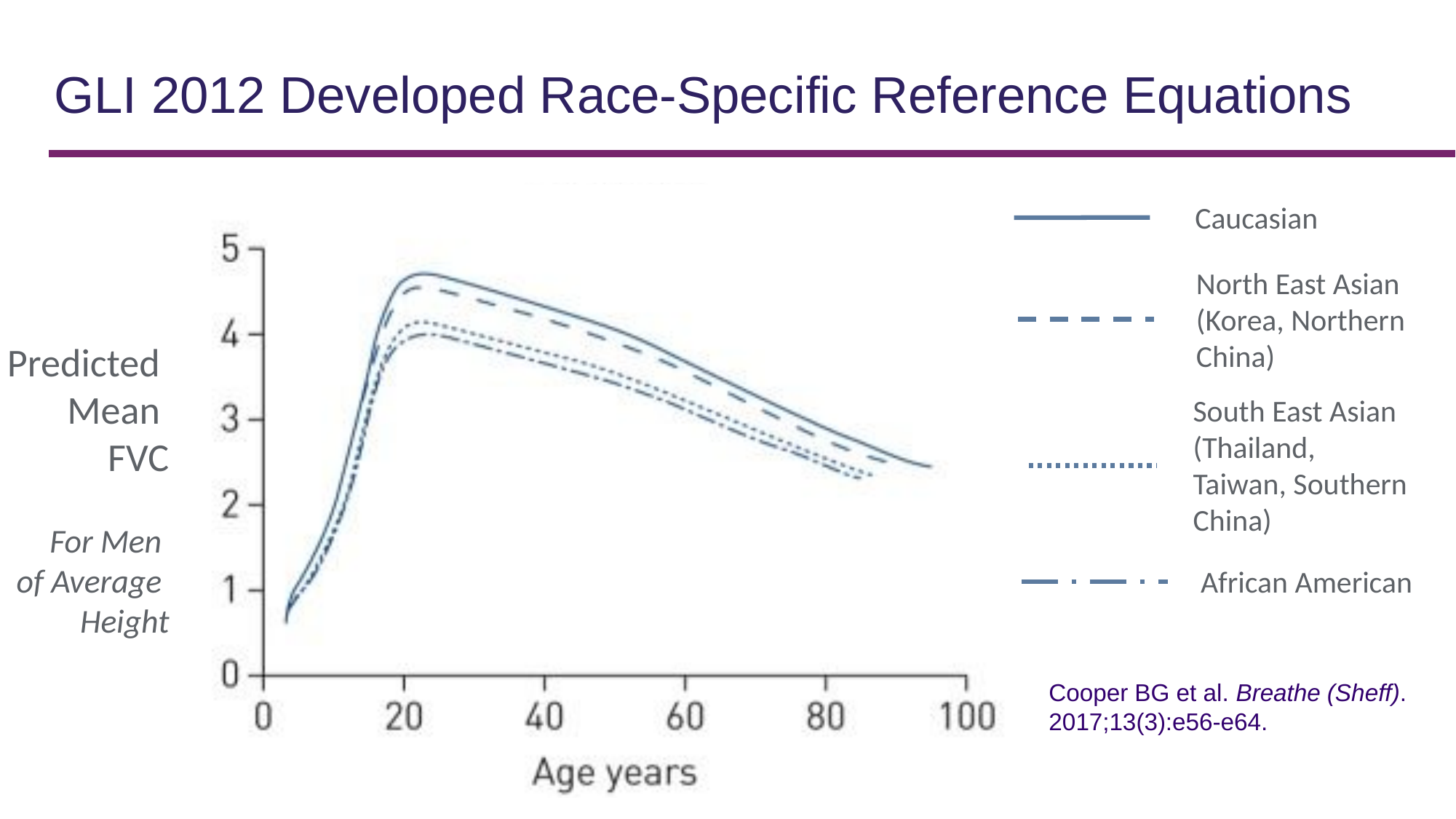

# GLI 2012 Developed Race-Specific Reference Equations
Caucasian
North East Asian (Korea, Northern China)
Predicted
Mean
FVC
For Men
of Average
Height
South East Asian (Thailand, Taiwan, Southern China)
African American
Cooper BG et al. Breathe (Sheff). 2017;13(3):e56-e64.

## Slide 19
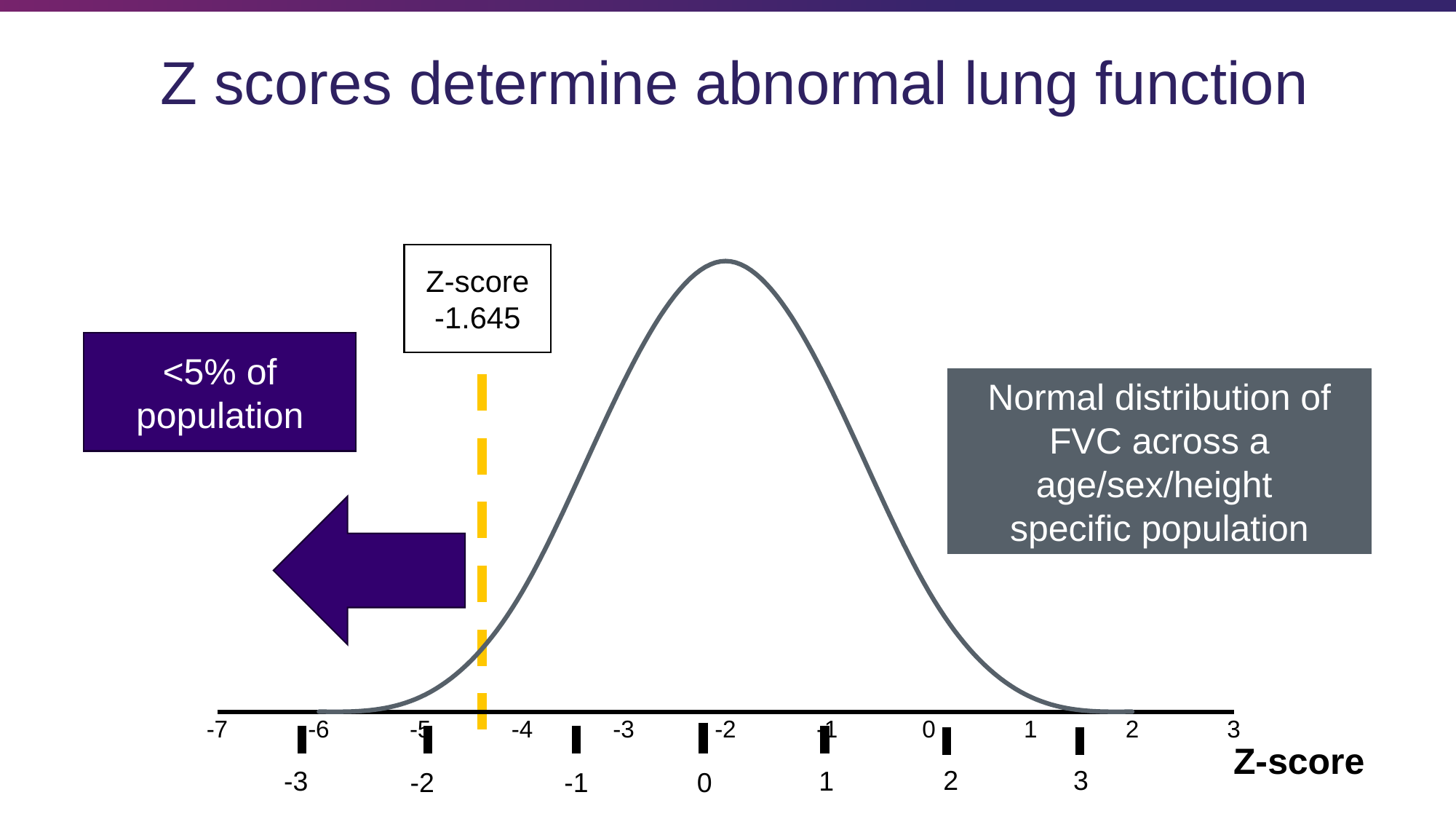

Z scores determine abnormal lung function
### Chart
| Category | |
|---|---|
### Chart
| Category | |
|---|---| Z-score
-1.645
<5% of population
Normal distribution of FVC across a age/sex/height
specific population
Z-score
2
3
-3
1
-2
-1
0

## Slide 20
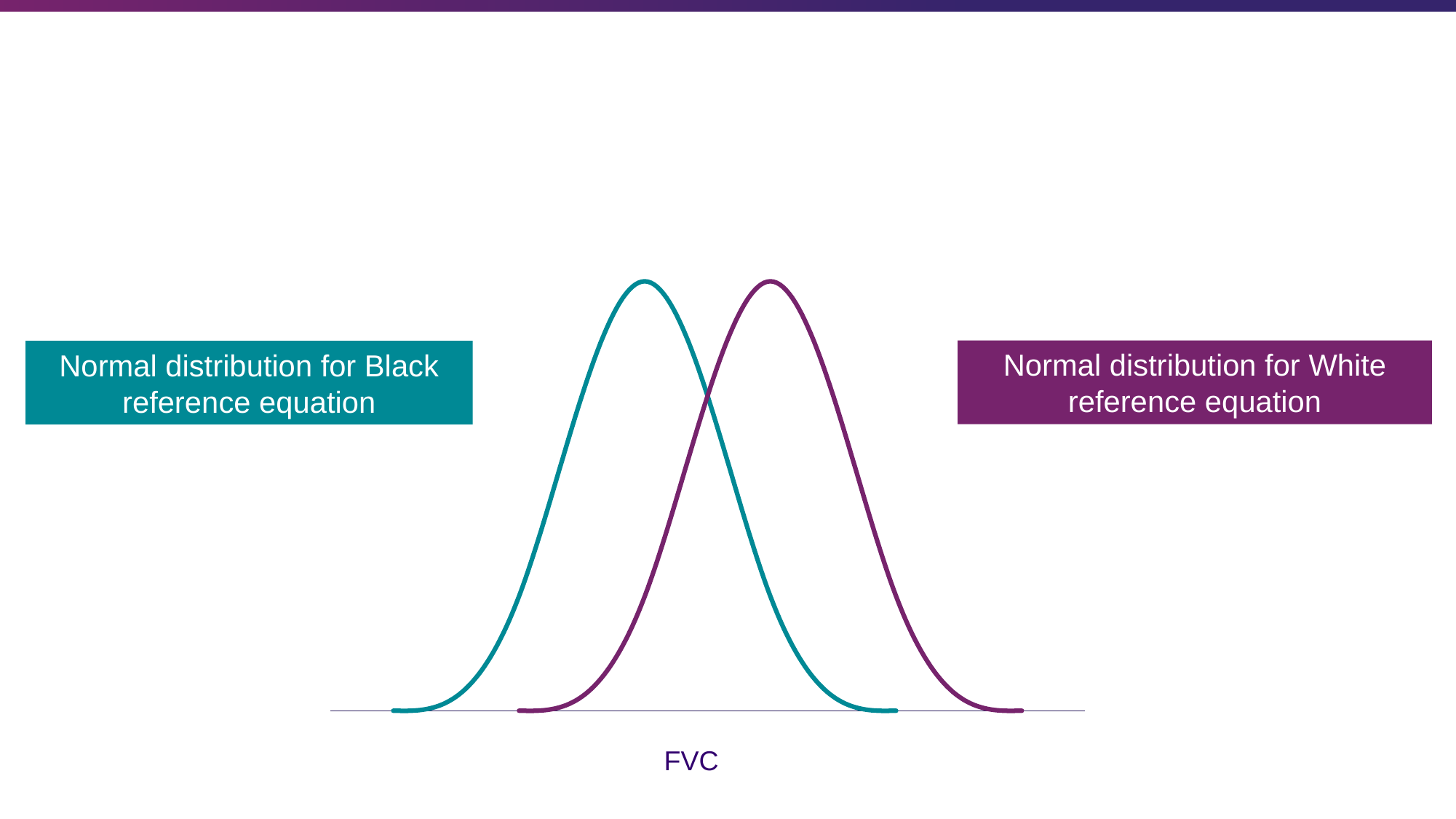

### Chart
| Category | | |
|---|---|---|Normal distribution for White reference equation
Normal distribution for Black reference equation
FVC

## Slide 21
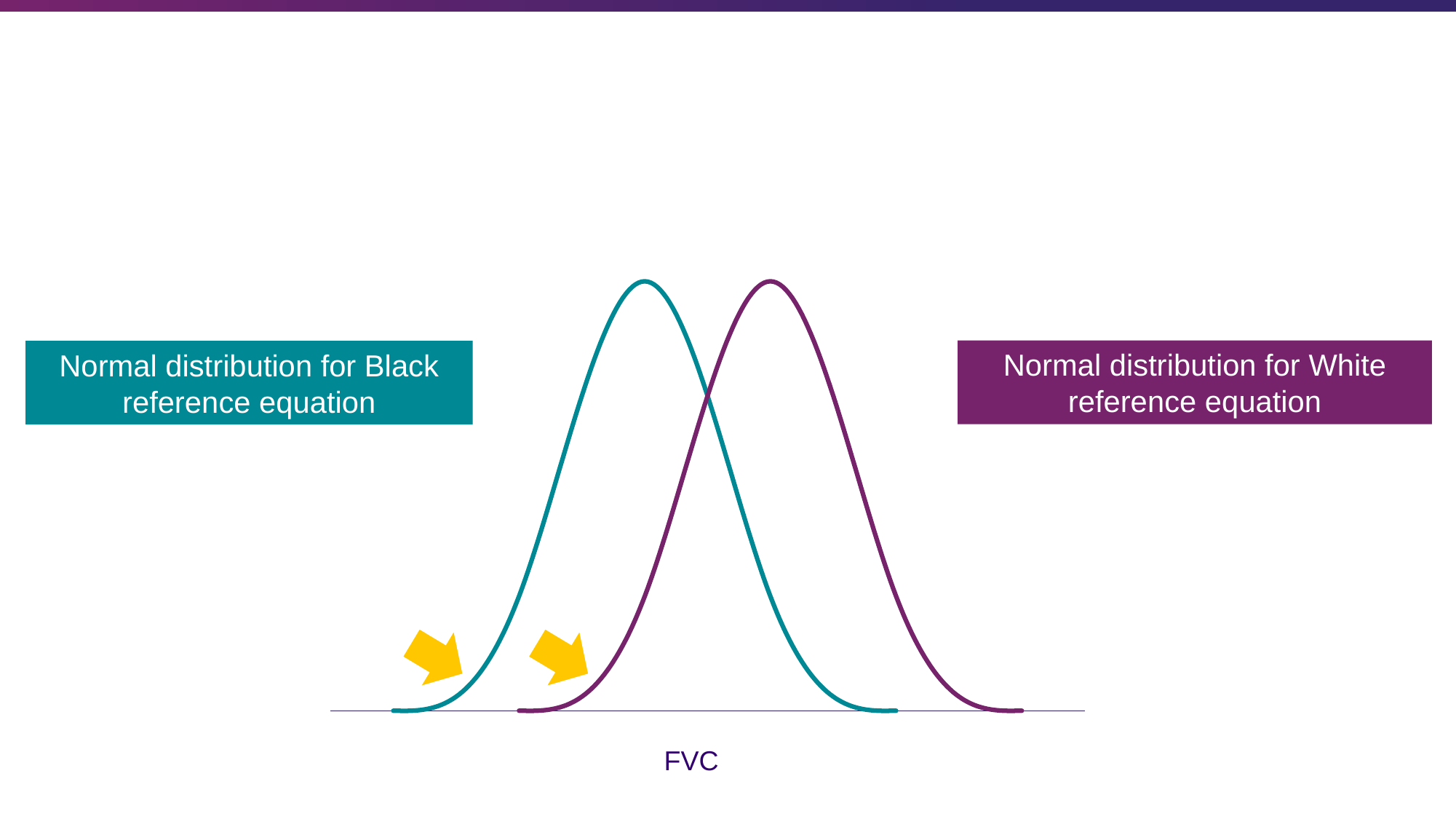

### Chart
| Category | | |
|---|---|---|Normal distribution for White reference equation
Normal distribution for Black reference equation
FVC

## Slide 22
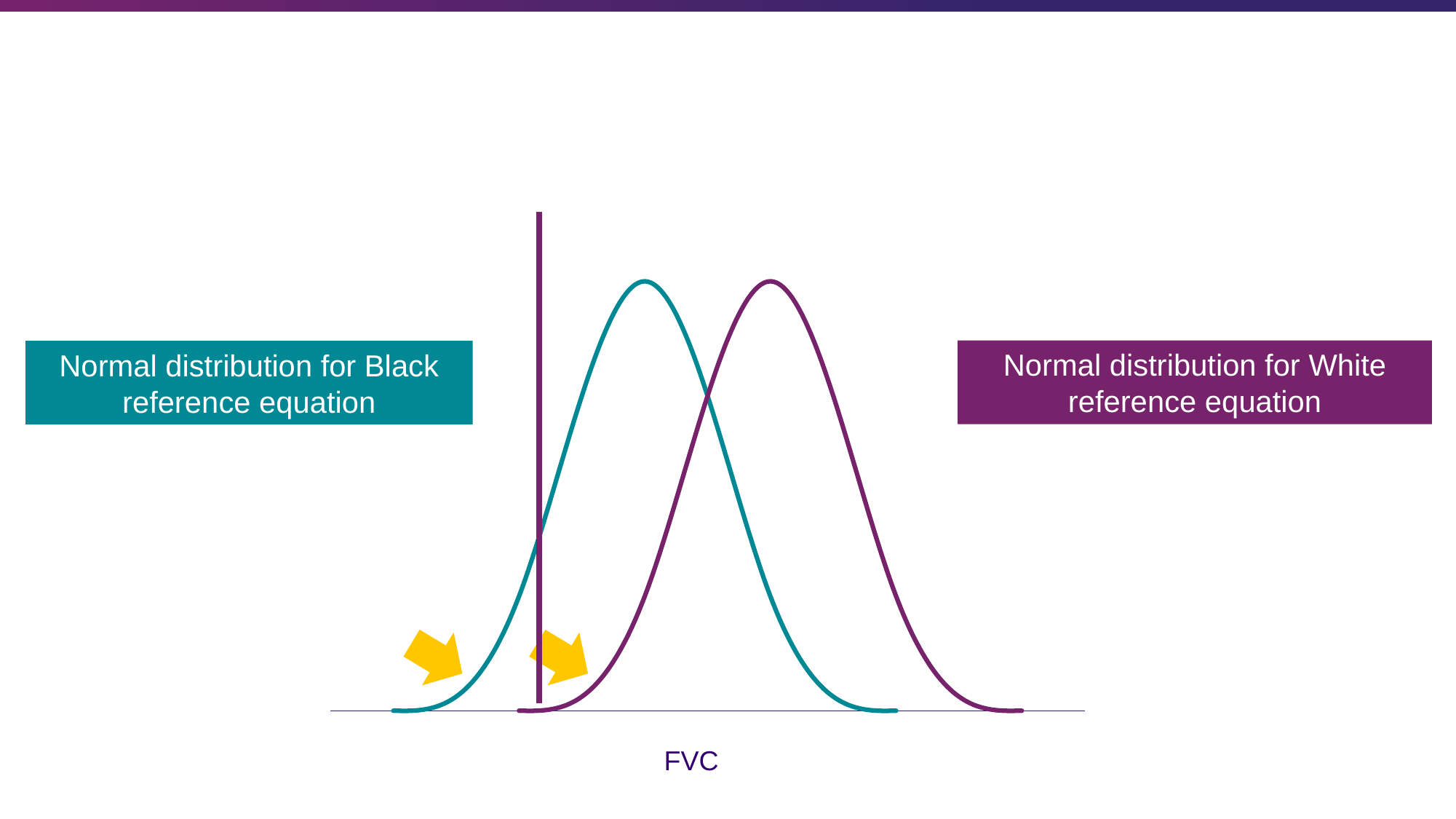

### Chart
| Category | | |
|---|---|---|Normal distribution for White reference equation
Normal distribution for Black reference equation
FVC

## Slide 23
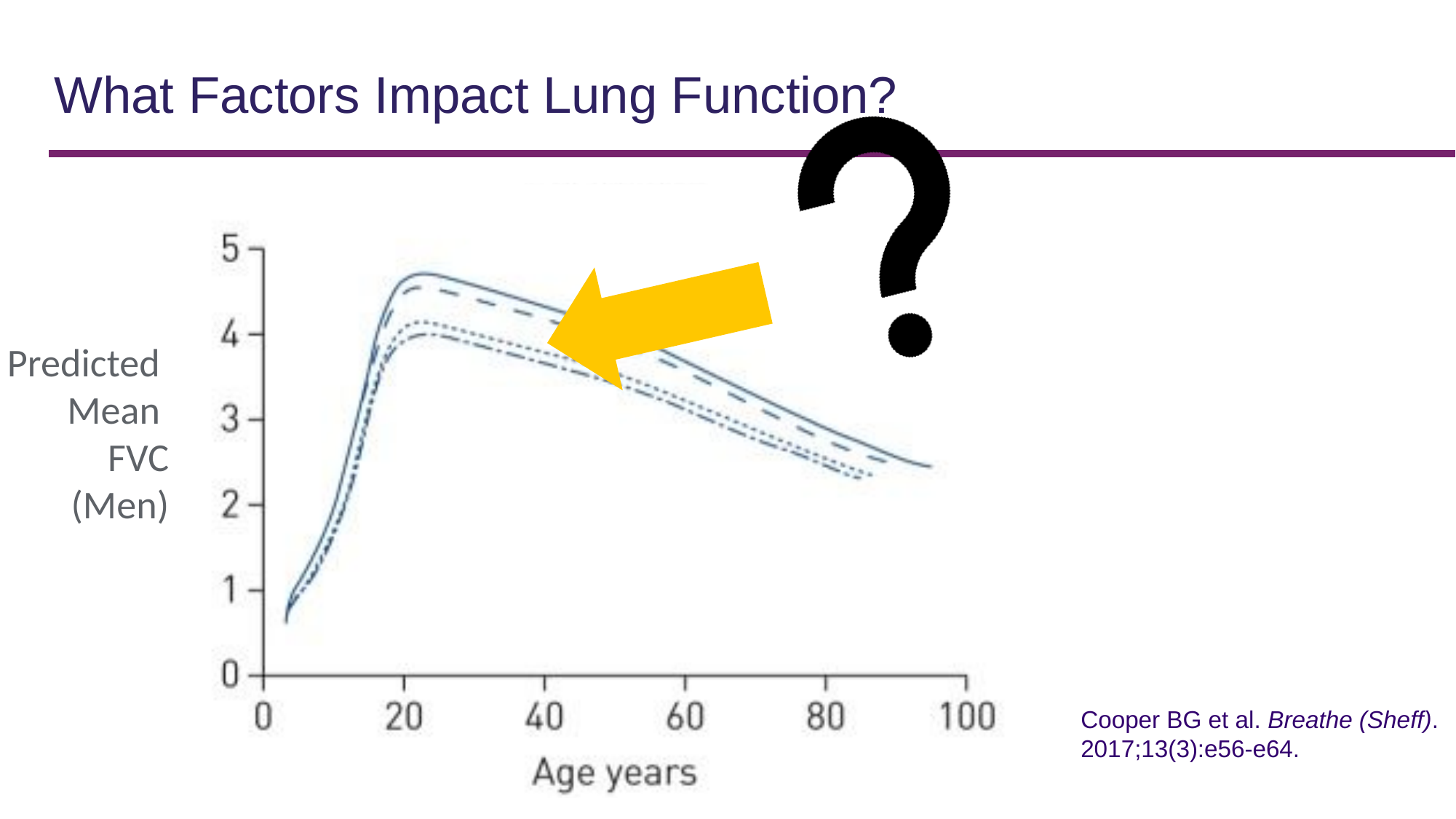

# What Factors Impact Lung Function?
Caucasian
North East Asian (Korea, Northern China)
Predicted
Mean
FVC
(Men)
South East Asian (Thailand, Taiwan, Southern China)
African American
Cooper BG et al. Breathe (Sheff). 2017;13(3):e56-e64.

## Slide 24
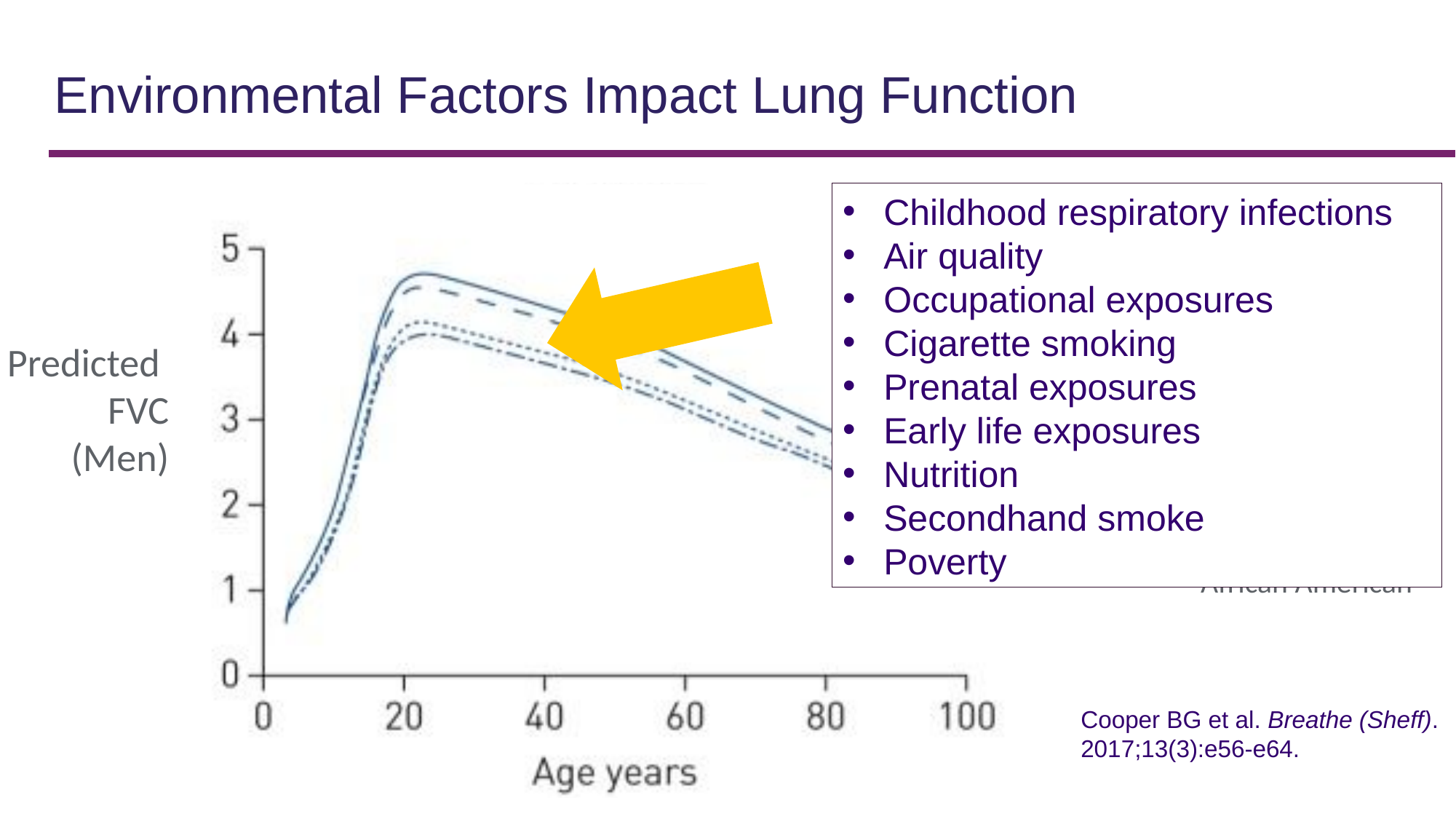

# Environmental Factors Impact Lung Function
Childhood respiratory infections
Air quality
Occupational exposures
Cigarette smoking
Prenatal exposures
Early life exposures
Nutrition
Secondhand smoke
Poverty
Caucasian
North East Asian (Korea, Northern China)
Predicted
FVC
(Men)
South East Asian (Thailand, Taiwan, Southern China)
African American
Cooper BG et al. Breathe (Sheff). 2017;13(3):e56-e64.

## Slide 25
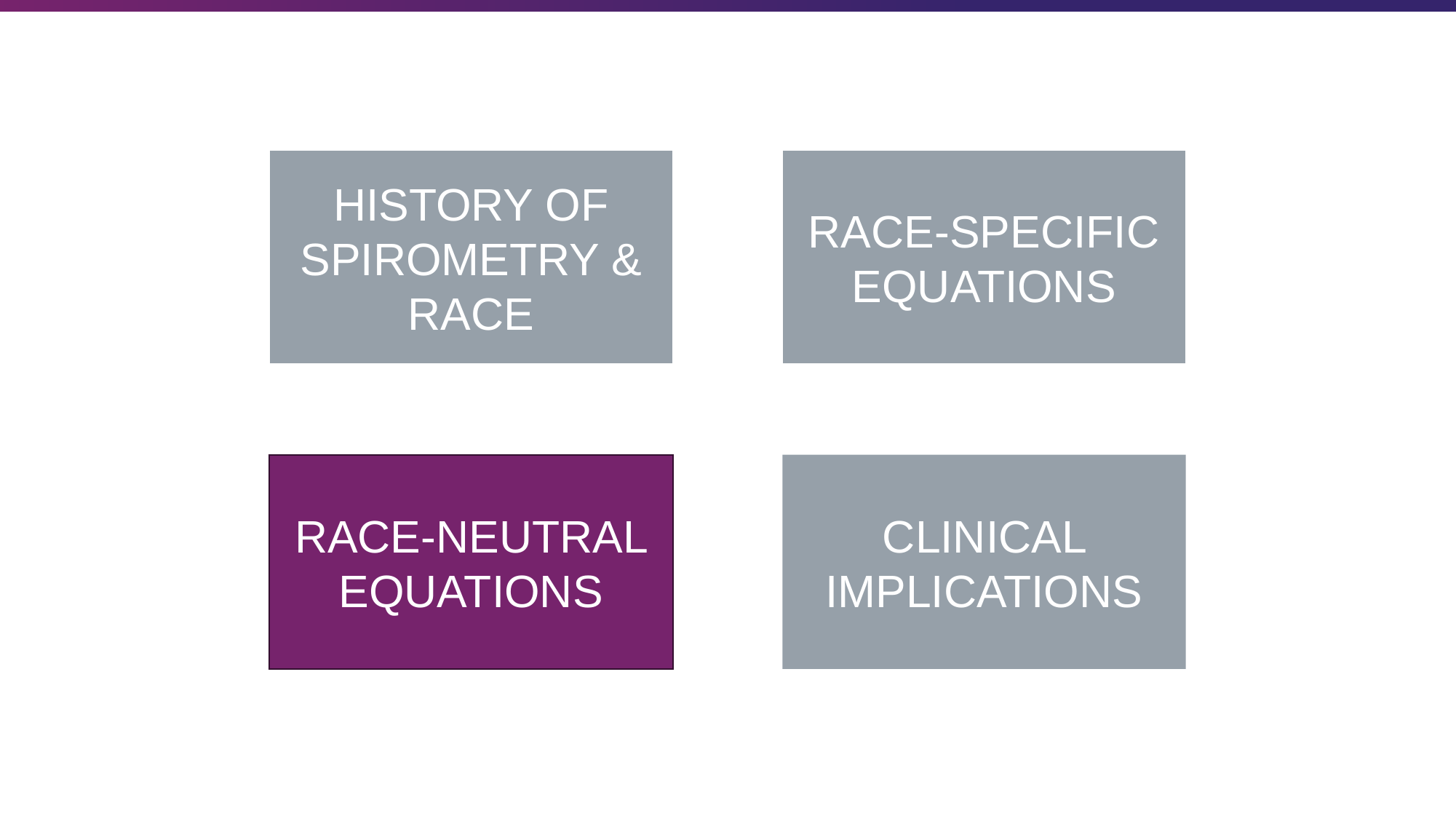

HISTORY OF SPIROMETRY & RACE
RACE-SPECIFIC EQUATIONS
RACE-NEUTRAL EQUATIONS
CLINICAL IMPLICATIONS

## Slide 26
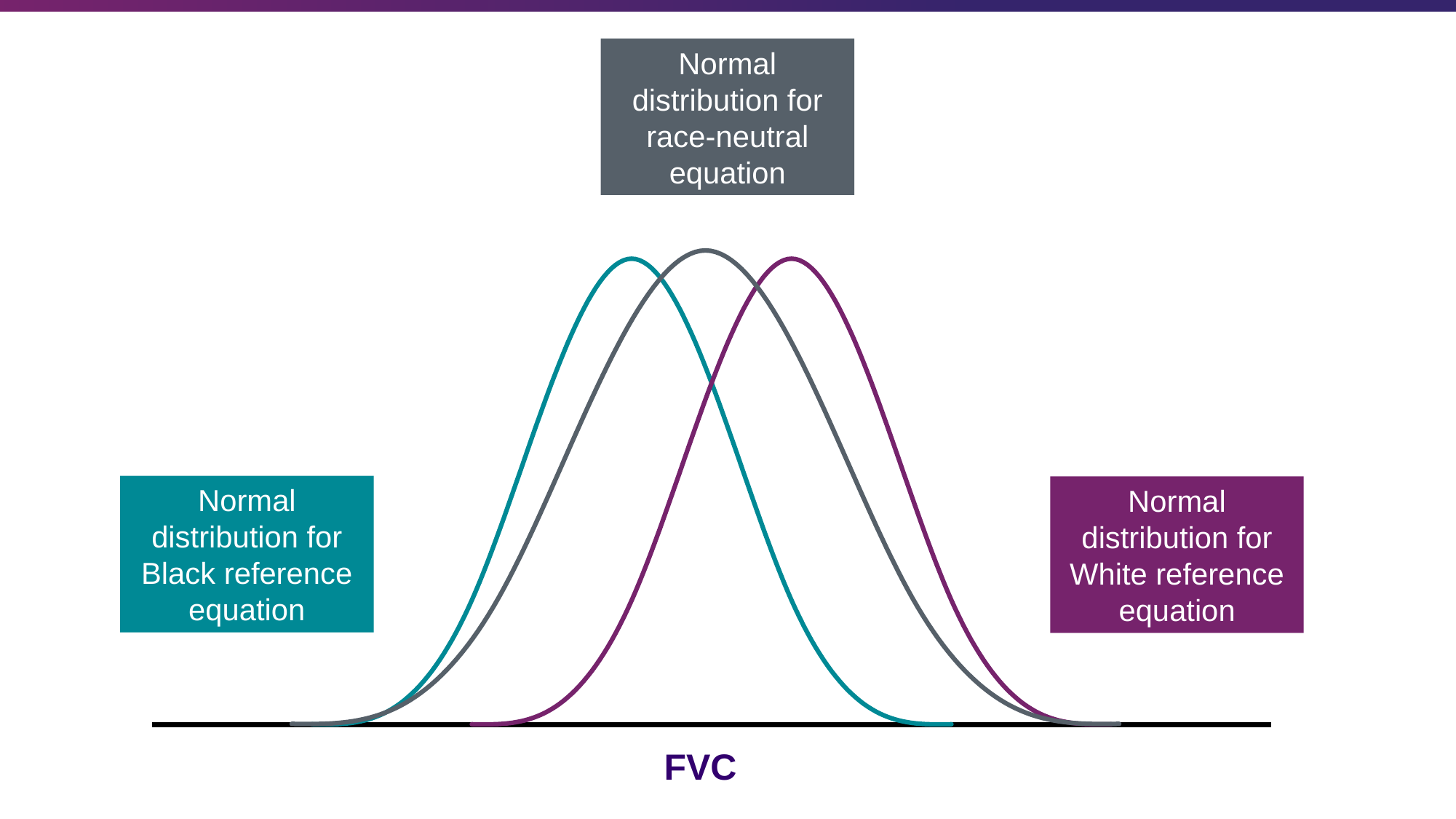

Normal distribution for race-neutral equation
### Chart
| Category | |
|---|---|
### Chart
| Category | | |
|---|---|---|Normal distribution for Black reference equation
Normal distribution for White reference equation
FVC

## Slide 27
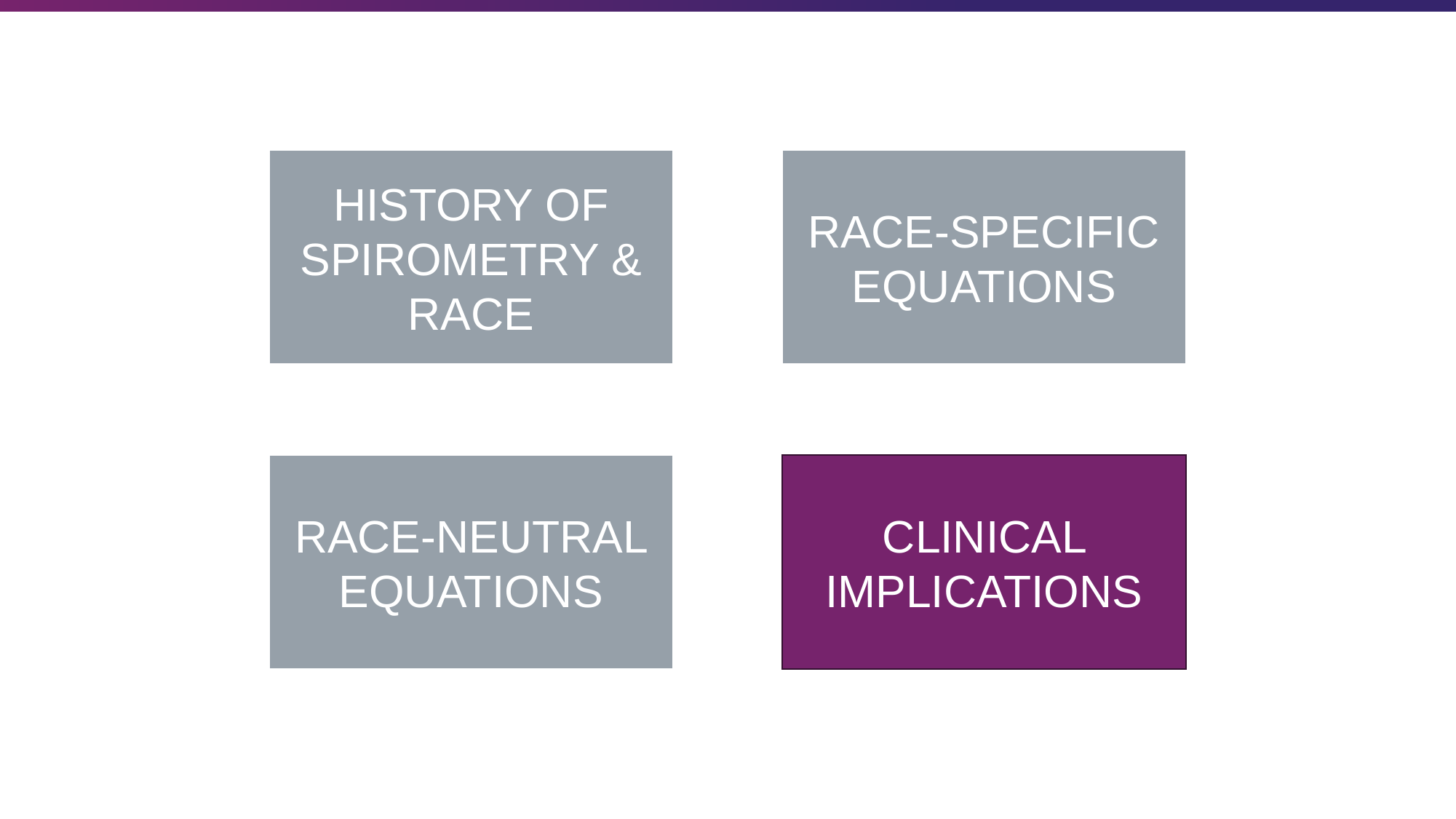

HISTORY OF SPIROMETRY & RACE
HISTORY OF SPIROMETRY
RACE-SPECIFIC EQUATIONS
RACE-SPECIFIC EQUATIONS
RACE-NEUTRAL EQUATIONS
RACE-NEUTRAL EQUATIONS
CLINICAL IMPLICATIONS

## Slide 28
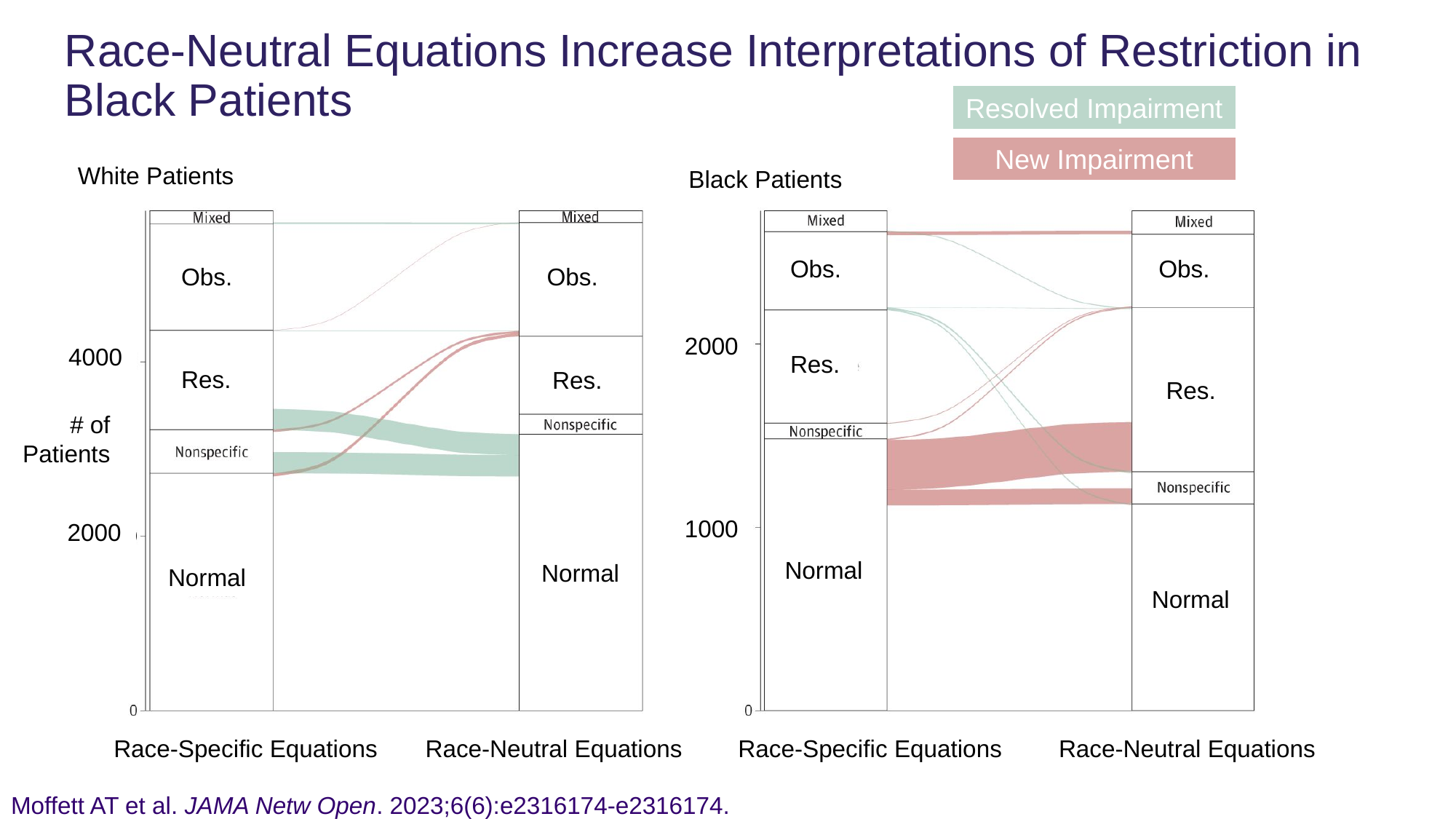

# Race-Neutral Equations Increase Interpretations of Restriction in Black Patients
Resolved Impairment
New Impairment
White Patients
Black Patients
Obs.
Obs.
Obs.
Obs.
2000
4000
Res.
Res.
Res.
Res.
# of Patients
1000
2000
Normal
Normal
Normal
Normal
Race-Specific Equations
Race-Neutral Equations
Race-Specific Equations
Race-Neutral Equations
Moffett AT et al. JAMA Netw Open. 2023;6(6):e2316174-e2316174.

## Slide 29
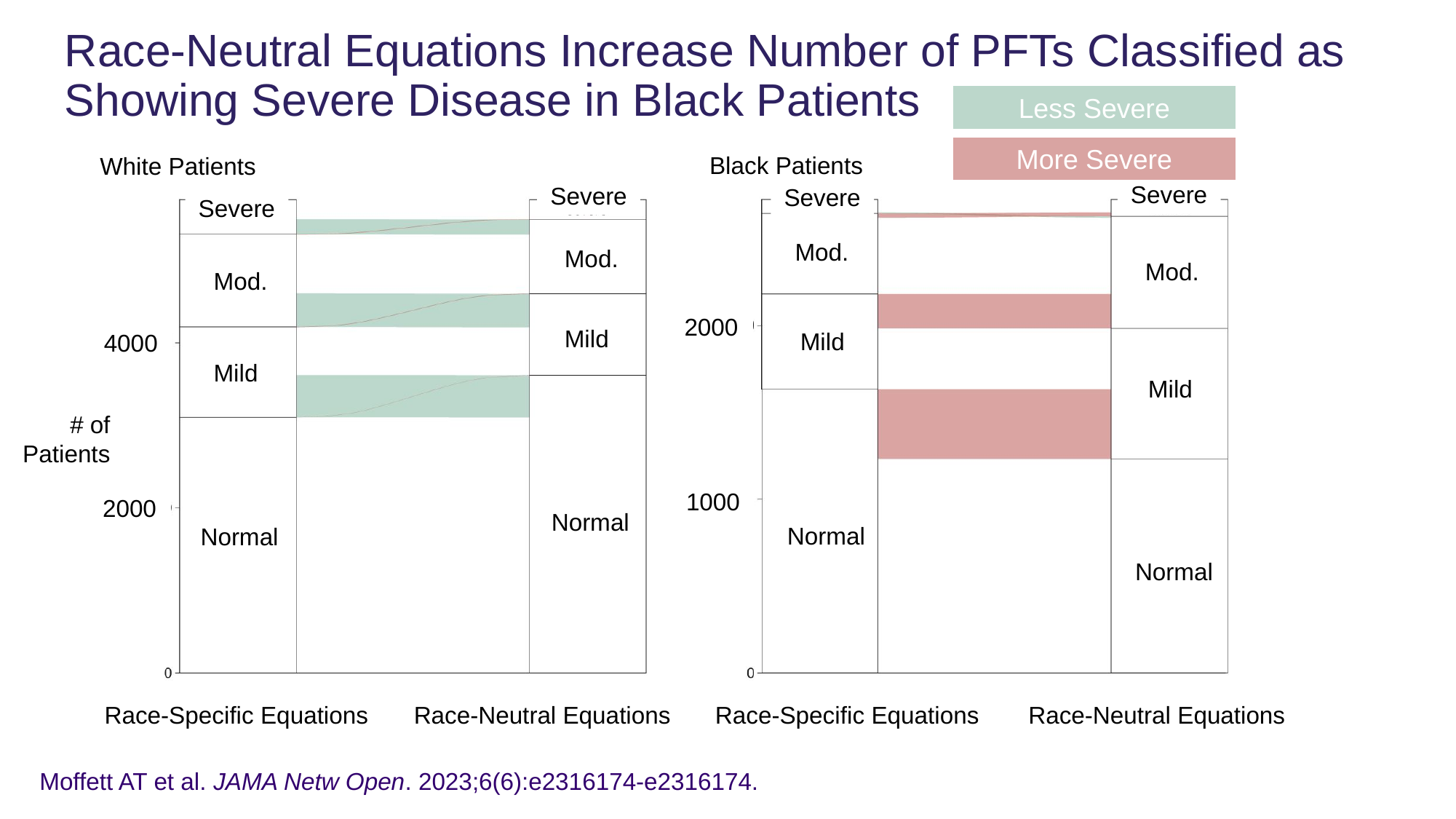

# Race-Neutral Equations Increase Number of PFTs Classified as Showing Severe Disease in Black Patients
Less Severe
More Severe
Black Patients
White Patients
Severe
Severe
Severe
Severe
Mod.
Mod.
Mod.
Mod.
2000
Mild
Mild
4000
Mild
Mild
# of Patients
1000
2000
Normal
Normal
Normal
Normal
Race-Specific Equations
Race-Neutral Equations
Race-Specific Equations
Race-Neutral Equations
Moffett AT et al. JAMA Netw Open. 2023;6(6):e2316174-e2316174.

## Slide 30
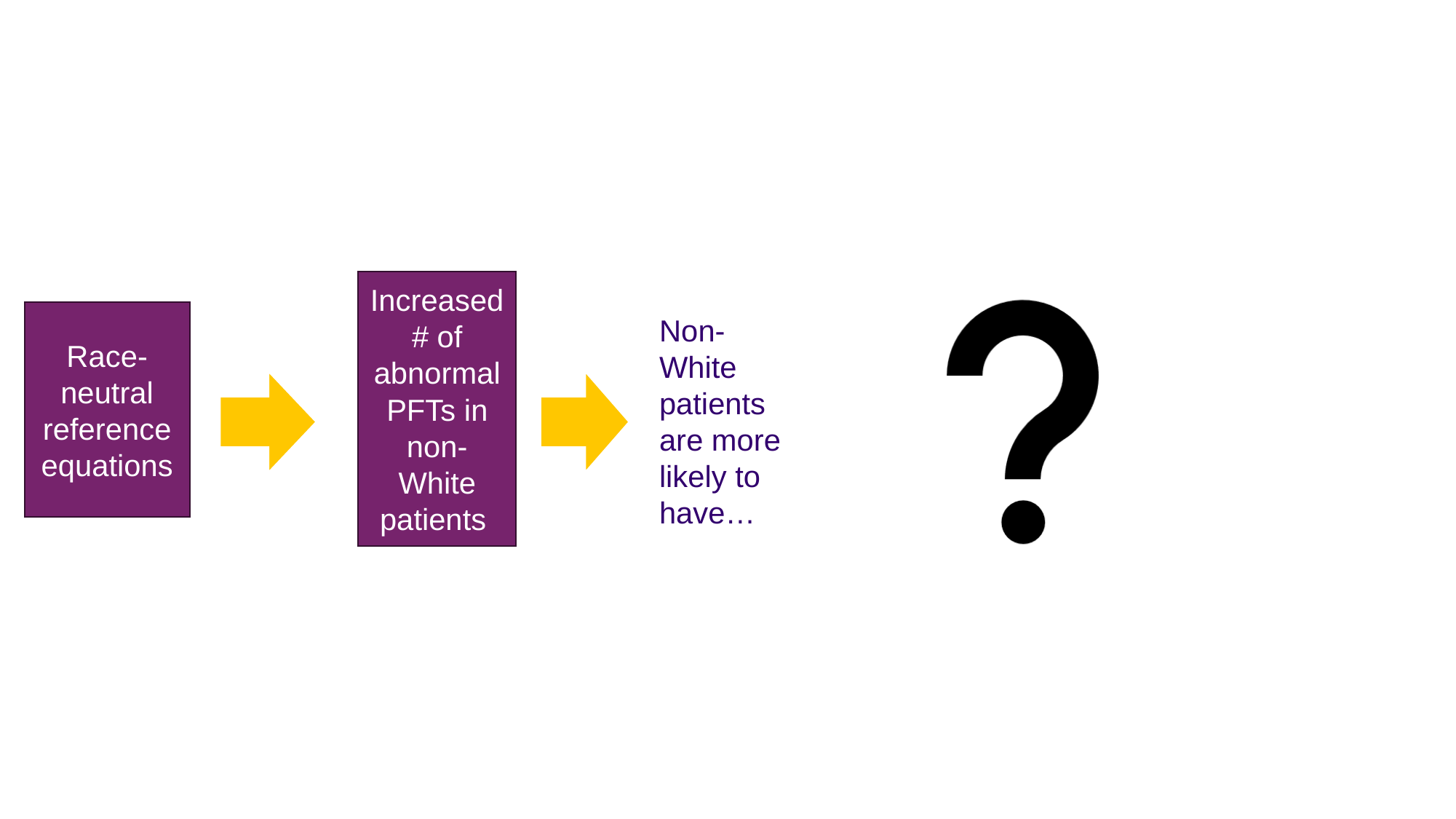

Increased # of abnormal PFTs in non-White patients
Race-neutral reference equations
Non-White patients are more likely to have…

## Slide 31
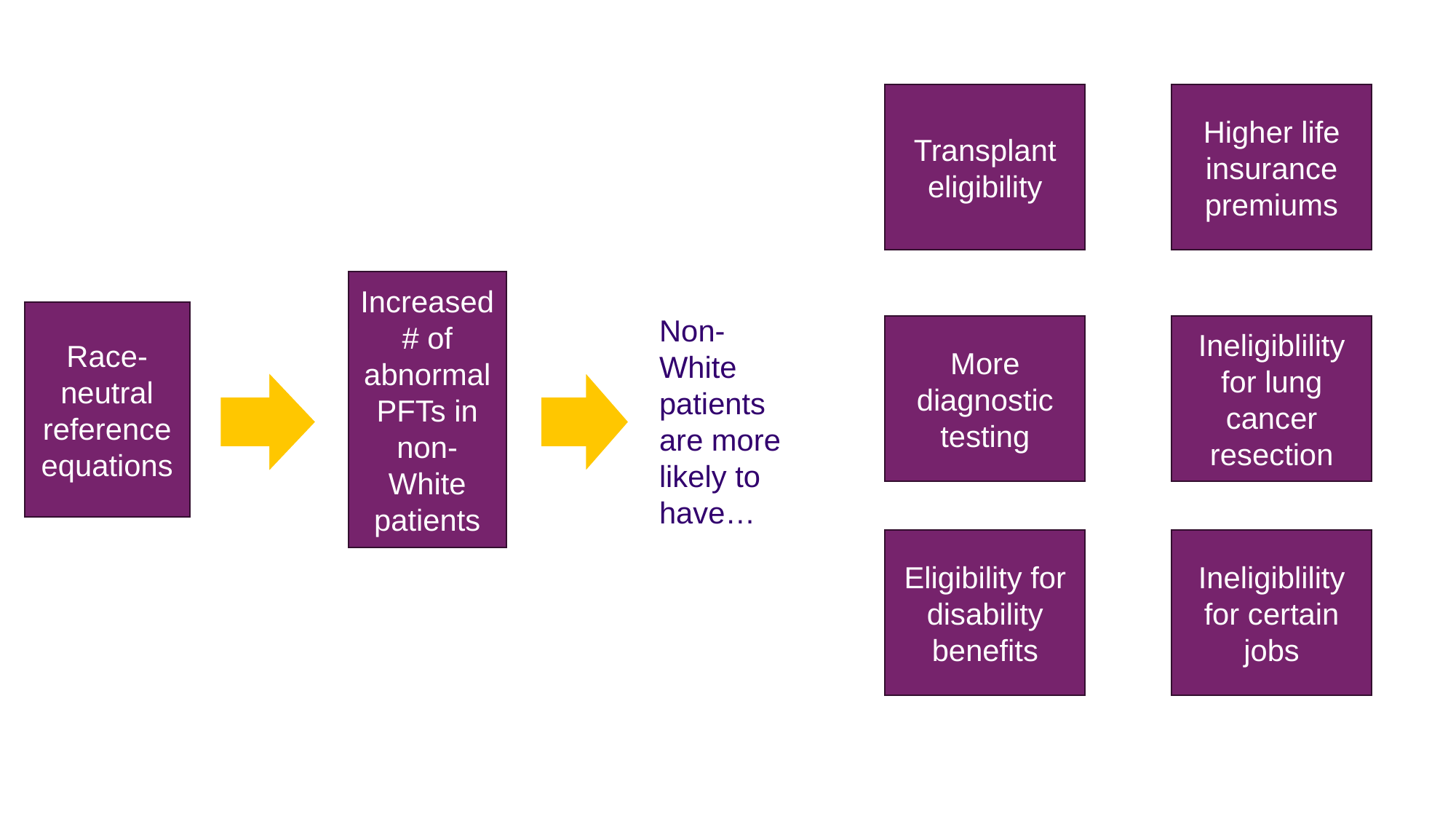

Transplant eligibility
Higher life insurance premiums
Increased # of abnormal PFTs in non-White patients
Race-neutral reference equations
Non-White patients are more likely to have…
Ineligiblility for lung cancer resection
More diagnostic testing
Eligibility for disability benefits
Ineligiblility for certain jobs

## Slide 32
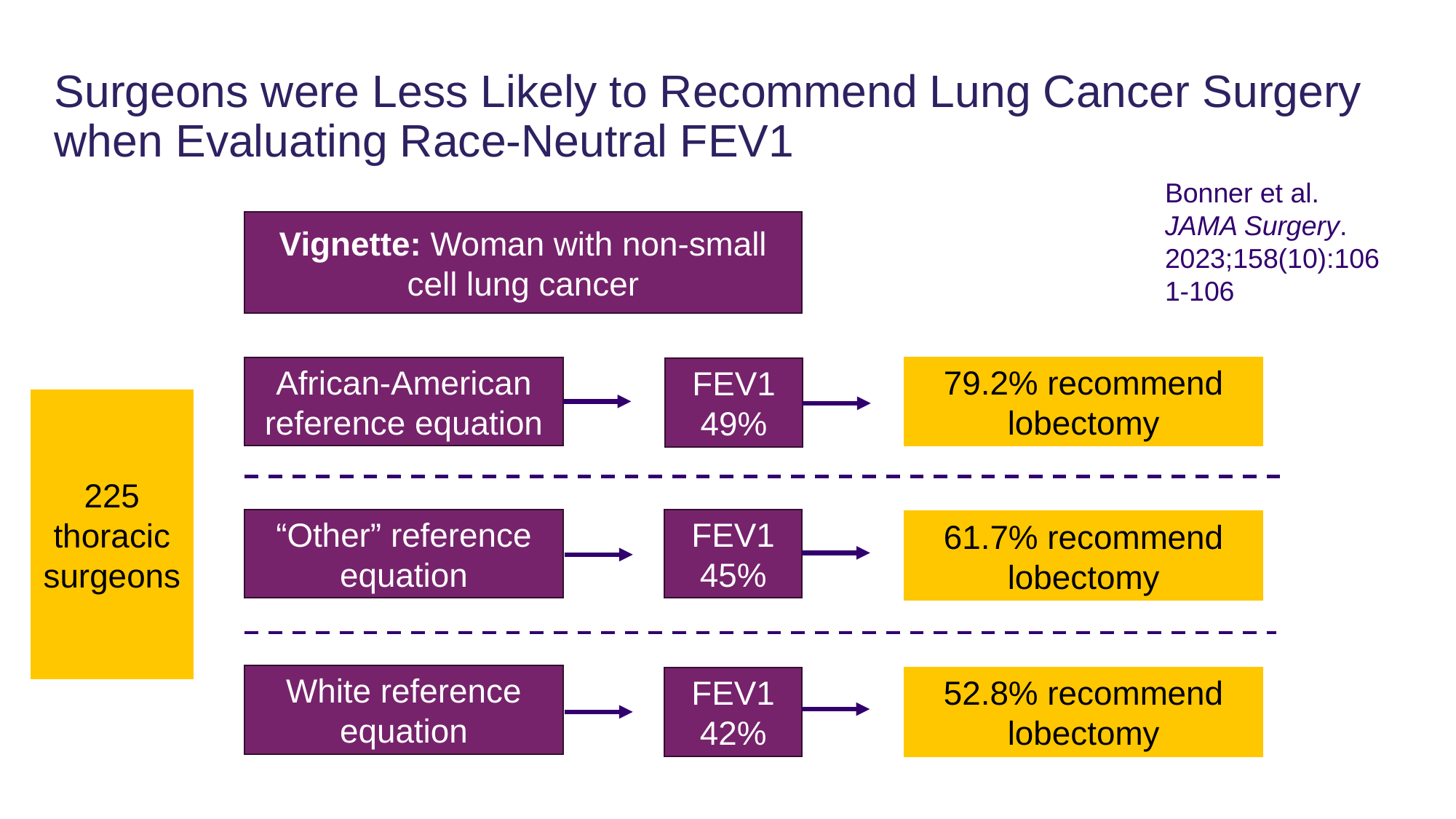

# Surgeons were Less Likely to Recommend Lung Cancer Surgery when Evaluating Race-Neutral FEV1
Bonner et al. JAMA Surgery. 2023;158(10):1061-106
Vignette: Woman with non-small cell lung cancer
African-American reference equation
FEV1 49%
“Other” reference equation
FEV1 45%
White reference equation
FEV1 42%
79.2% recommend lobectomy
225 thoracic surgeons
61.7% recommend lobectomy
52.8% recommend lobectomy

## Slide 33
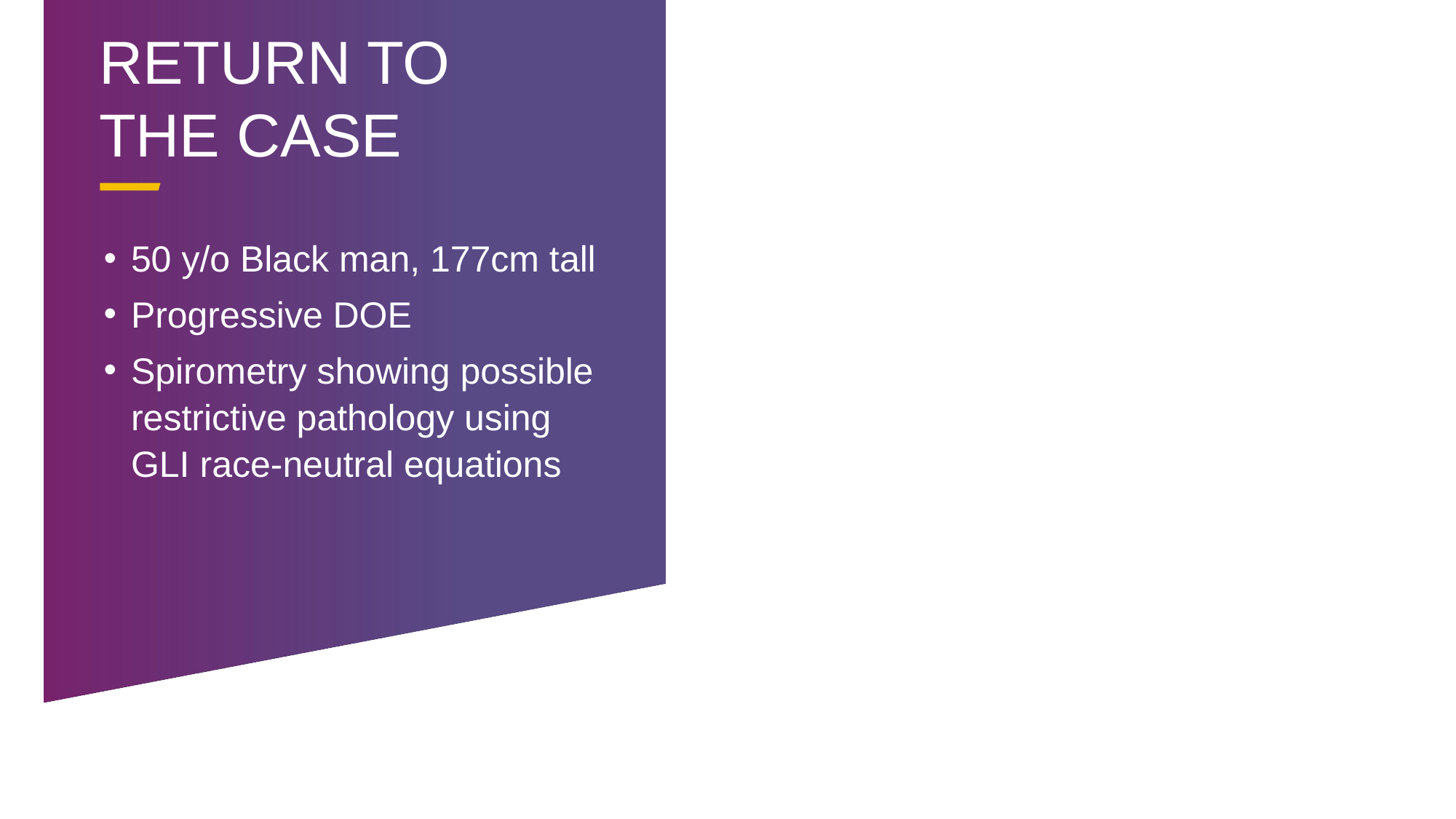

RETURN TO THE CASE
50 y/o Black man, 177cm tall
Progressive DOE
Spirometry showing possible restrictive pathology using GLI race-neutral equations

## Slide 34
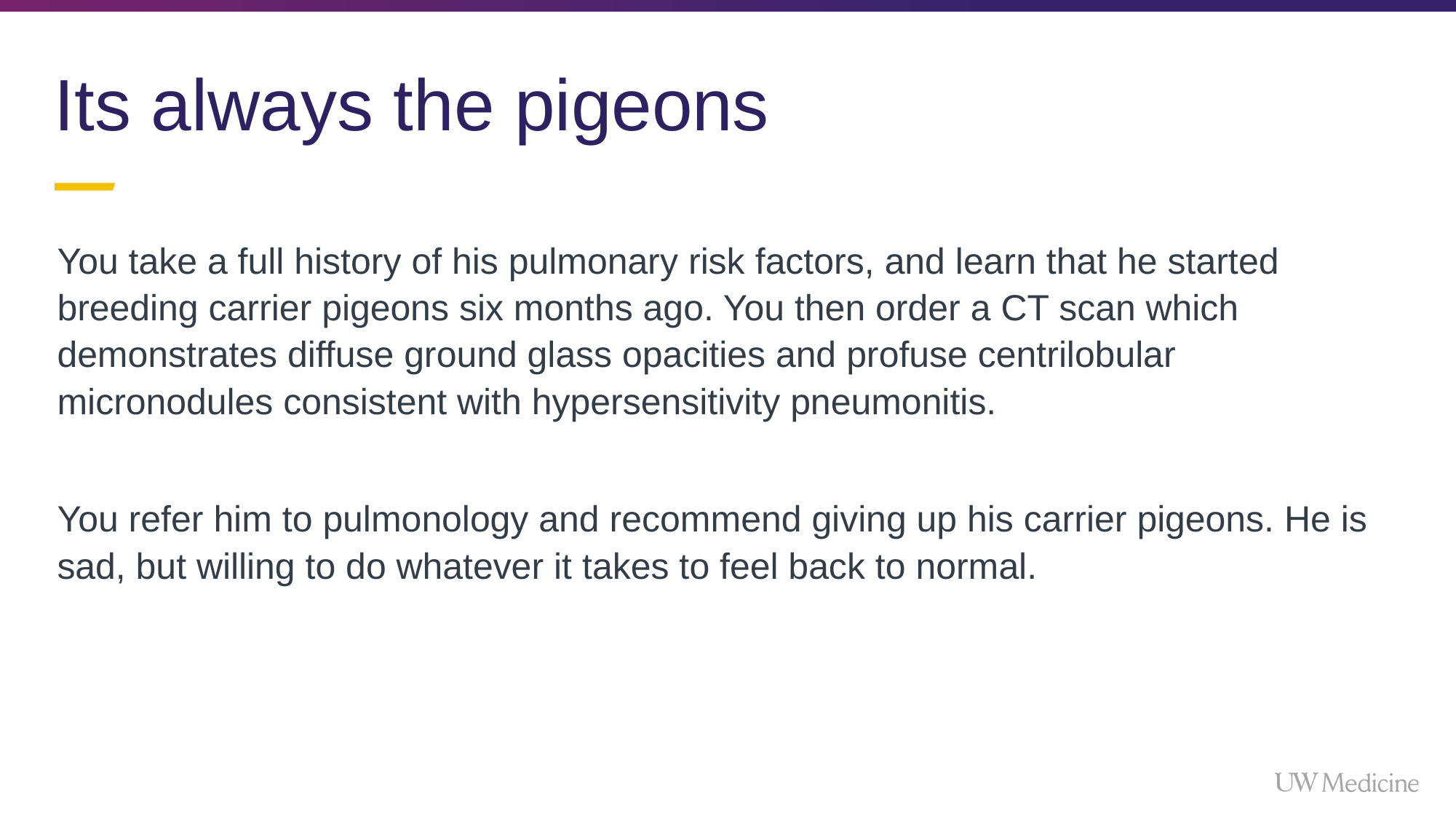

# Its always the pigeons
You take a full history of his pulmonary risk factors, and learn that he started breeding carrier pigeons six months ago. You then order a CT scan which demonstrates diffuse ground glass opacities and profuse centrilobular micronodules consistent with hypersensitivity pneumonitis.
You refer him to pulmonology and recommend giving up his carrier pigeons. He is sad, but willing to do whatever it takes to feel back to normal.

## Slide 35
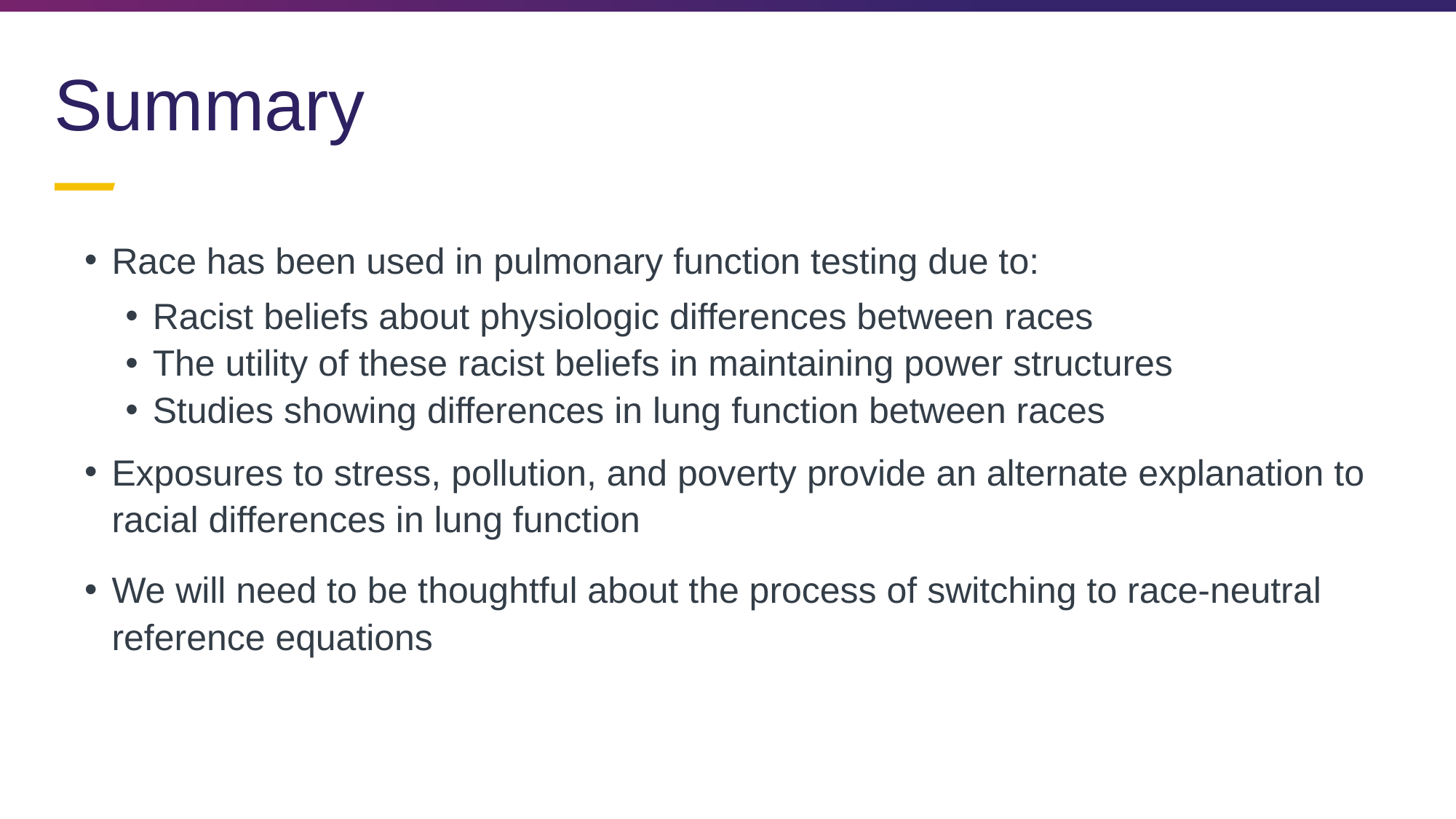

# Summary
Race has been used in pulmonary function testing due to:
Racist beliefs about physiologic differences between races
The utility of these racist beliefs in maintaining power structures
Studies showing differences in lung function between races
Exposures to stress, pollution, and poverty provide an alternate explanation to racial differences in lung function
We will need to be thoughtful about the process of switching to race-neutral reference equations

## Slide 36
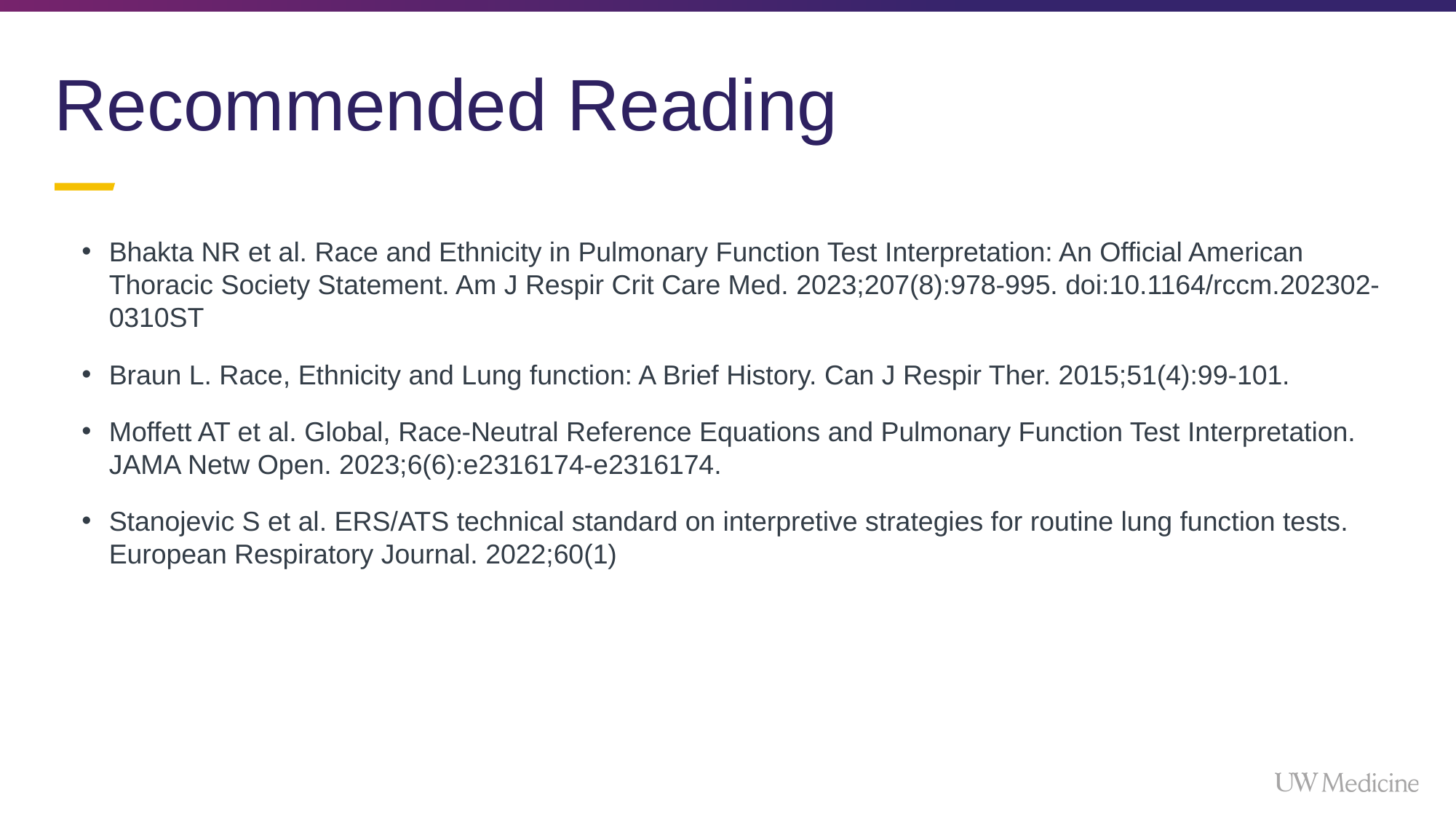

# Recommended Reading
Bhakta NR et al. Race and Ethnicity in Pulmonary Function Test Interpretation: An Official American Thoracic Society Statement. Am J Respir Crit Care Med. 2023;207(8):978-995. doi:10.1164/rccm.202302-0310ST
Braun L. Race, Ethnicity and Lung function: A Brief History. Can J Respir Ther. 2015;51(4):99-101.
Moffett AT et al. Global, Race-Neutral Reference Equations and Pulmonary Function Test Interpretation. JAMA Netw Open. 2023;6(6):e2316174-e2316174.
Stanojevic S et al. ERS/ATS technical standard on interpretive strategies for routine lung function tests. European Respiratory Journal. 2022;60(1)

## Slide 37
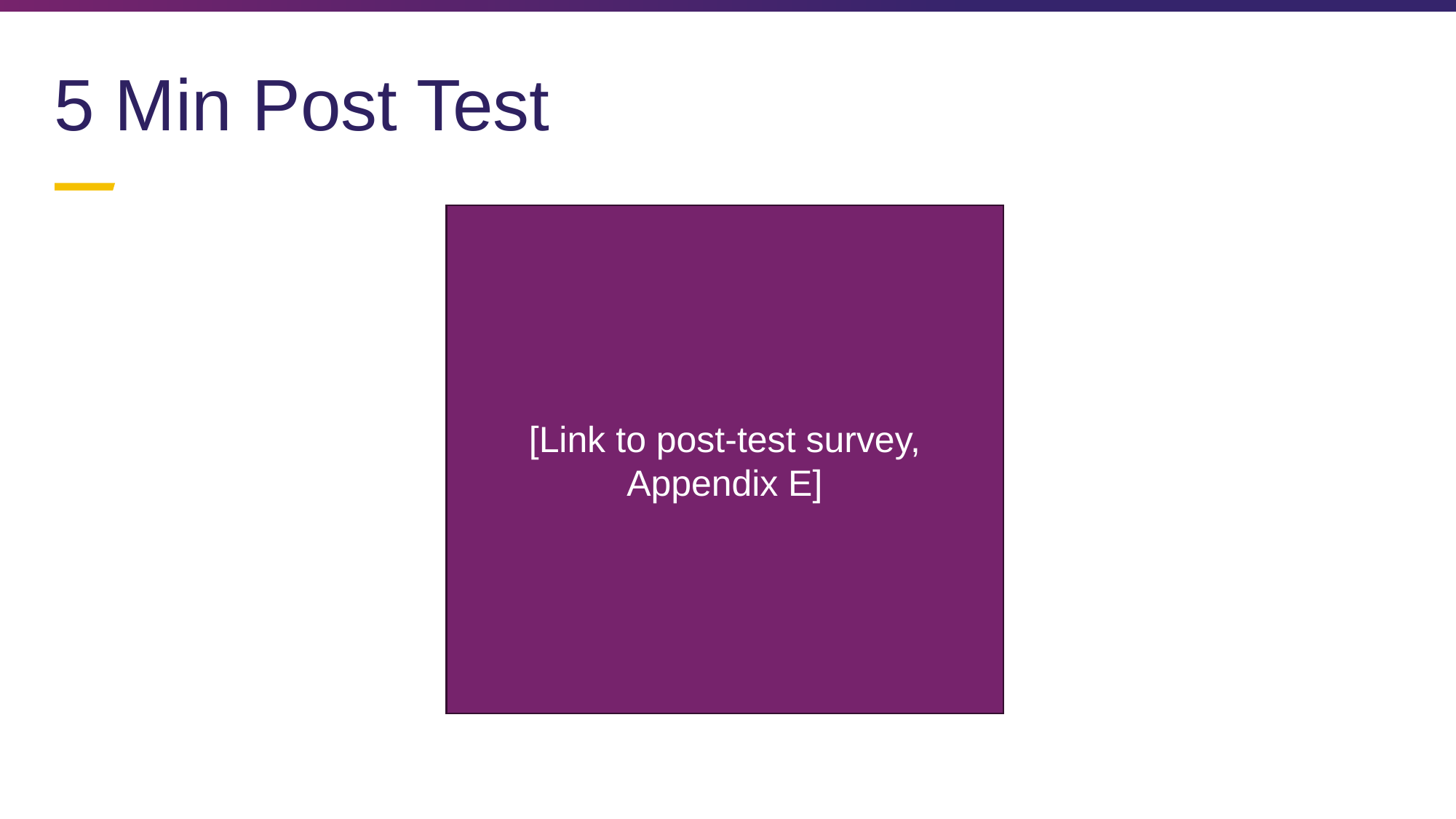

# 5 Min Post Test
[Link to post-test survey, Appendix E]
